# Image‐guided metabolomics and transcriptomics reveal tumour heterogeneity in luminal A and B human breast cancer beyond glucose tracer uptake

**DOI:** 10.1002/ctm2.1550

**Published:** 2024-02-08

**Authors:** Qianlu Yang, Sisi Deng, Heike Preibsch, Tim‐Colin Schade, André Koch, Georgy Berezhnoy, Laimdota Zizmare, Anna Fischer, Brigitte Gückel, Annette Staebler, Andreas D. Hartkopf, Bernd J. Pichler, Christian la Fougère, Markus Hahn, Irina Bonzheim, Konstantin Nikolaou, Christoph Trautwein

**Affiliations:** ^1^ Department of Preclinical Imaging and Radiopharmacy Werner Siemens Imaging Center University Hospital Tuebingen Tuebingen Germany; ^2^ Cluster of Excellence iFIT (EXC 2180) “Image Guided and Functionally Instructed Tumor Therapies” University of Tuebingen Tuebingen Germany; ^3^ Department of Diagnostic and Interventional Radiology University Hospital Tuebingen Tuebingen Germany; ^4^ Department of Pathology and Neuropathology University Hospital Tuebingen Tuebingen Germany; ^5^ Department of Women's Health University Hospital Tuebingen Tuebingen Germany; ^6^ German Cancer Research Center German Cancer Consortium DKTK Partner Site Tuebingen Tuebingen Germany; ^7^ Department of Nuclear Medicine and Clinical Molecular Imaging University Hospital Tuebingen Tuebingen Germany

**Keywords:** [^18^F]FDG, ^1^H‐NMR, gene expression, PET/MR, tumour microenvironment, multi‐omics

## Abstract

**Background:**

Breast cancer is a metabolically heterogeneous disease, and although the concept of heterogeneous cancer metabolism is known, its precise role in human breast cancer is yet to be fully elucidated.

**Methods:**

We investigated in an explorative approach a cohort of 42 primary mamma carcinoma patients with positron emission tomography/magnetic resonance imaging (PET/MR) prior to surgery, followed by histopathology and molecular diagnosis. From a subset of patients, which showed high metabolic heterogeneity based on tracer uptake and pathology classification, tumour centre and periphery specimen tissue samples were further investigated by a targeted breast cancer gene expression panel and quantitative metabolomics by nuclear magnetic resonance (NMR) spectroscopy. All data were analysed in a combinatory approach.

**Results:**

[^18^F]FDG (2‐deoxy‐2‐[fluorine‐18]fluoro‐d‐glucose) tracer uptake confirmed dominance of glucose metabolism in the breast tumour centre, with lower levels in the periphery. Additionally, we observed differences in lipid and proliferation related genes between luminal A and B subtypes in the centre and periphery. Tumour periphery showed elevated acetate levels and enrichment in lipid metabolic pathways genes especially in luminal B. Furthermore, serine was increased in the periphery and higher expression of thymidylate synthase (TYMS) indicated one‐carbon metabolism increased in tumour periphery. The overall metabolic activity based on [^18^F]FDG uptake of luminal B subtype was higher than that of luminal A and the difference between the periphery and centre increased with tumour grade.

**Conclusion:**

Our analysis indicates variations in metabolism among different breast cancer subtypes and sampling locations which details the heterogeneity of the breast tumours. Correlation analysis of [^18^F]FDG tracer uptake, transcriptome and tumour metabolites like acetate and serine facilitate the search for new candidates for metabolic tracers and permit distinguishing luminal A and B. This knowledge may help to differentiate subtypes preclinically or to provide patients guide for neoadjuvant therapy and optimised surgical protocols based on individual tumour metabolism.

## Graphical abstract

- Central regions of LumA and LumB are dominated by glucose metabolism.
- In contrast, peripheral regions of LumA and LumB exhibit mostly lipid and one‐carbon metabolism.
- Region‐ and tumour‐specific metabolites may aid in new metabolic tracers development.
- Differences in central and peripheral [^18^F]FDG glucose tracer uptake widen with tumour grade in LumA and LumB.

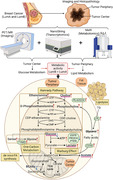

## 1 INTRODUCTION

Breast cancer is a heterogeneous disease where the tumour microenvironment (TME), genetic mutations and metabolic phenotypes play significant roles in its development.^1^ Global gene expression analyses have revealed the existence of at least five intrinsic subtypes of breast cancer (luminal A [LumA], luminal B [LumB], human epidermal growth factor receptor 2 [HER2]‐enriched and basal‐like), as well as a normal‐like group.^2^ These subtypes differ significantly in risk factors, incidence, baseline prognoses and responses to systemic therapies.^3^ Oestrogen receptor (ER) positive breast cancers are the most commonly occurring type and can be classified into two major molecular subtypes, LumA and LumB. Clinical manifestations, levels of malignancy, systematic therapeutic responses and survival outcomes can differ significantly between LumA and LumB despite similar histopathological features.^4^ Some molecular markers, such as progesterone receptor (PR), HER2 and Ki‐67 are commonly used to distinguish between these two types. Furthermore, LumB tumours have a faster proliferation rate and lower expression of PRs than LumA tumours, which by contrast, are associated with a higher cumulative metastasis rate.

Generally, cancer cells proliferate uncontrollably and have developed various strategies to maintain their proliferation rate high even under nutrient‐limited conditions, known as metabolic plasticity.^5^ Gene mutations, such as those affecting *p53*, can significantly alter tumour metabolism, leading to a shift towards a more glycolytic phenotype characterised by increased aerobic glycolysis and decreased typical glucose metabolism.^6^ Mutations in phosphatidylinositol‐4,5‐bisphosphate 3‐kinase catalytic subunit alpha (*PIK3CA*) activate the PI3K/AKT/mTOR pathway and are linked to increased glucose uptake, glycolysis and altered lipid metabolism.^7^ The high expression level of c‐Myc promotes aerobic glycolysis and lactate production. It also influences the one‐carbon unit synthesis pathway by up‐regulating key enzymes like 3‐phosphoglycerate dehydrogenase, phosphoserine aminotransferase and phosphoserine phosphatase.^8^, ^9^ Additionally, it drives glutamine metabolism by increasing the expression of glutamine transporters and glutaminase.^10^ Carbohydrate metabolism—including aerobic glycolysis, pentose phosphate pathways, tricarboxylic acid cycle and gluconeogenesis – is a major source of energy for breast cancer cells and can be dysregulated through various channels and pathways, thereby promoting proliferation and survival.^11^ In recent years, dysfunctional lipid metabolism has increasingly been recognised as a hallmark of cancer.^12^, ^13^ Clinical data indicate that postmenopausal women who are obese have a higher risk of developing breast cancer than lean women and is also associated with a poorer prognosis for women of all age groups.^14^, ^15^ In lipid metabolism, de novo fatty acid (FA) synthesis, FA uptake and transport and FA oxidation (FAO) are up‐regulated to meet the increased demand for biomass production.^16^ Furthermore, altered amino acid metabolism can be frequently observed in cancer. Amino acids serve as the primary building blocks of proteins, but can also regulate metabolites that support the growth of breast cancer cells.^17^

Utilising techniques like radiographic imaging and genomics, we can observe tumour heterogeneity, and sequencing biopsied tissues from various regions uncovers clonal amplification,^18^, ^19^ underscoring the challenge in cancer prevention and treatment. The uneven distribution of vasculature among tissues poses difficulties for effective therapeutic interventions. The TME, hosting diverse cell types, plays a pivotal role in shaping the unique metabolic landscape.^20^ Non‐tumour cells, including stromal and immune cells, actively contribute to the extracellular matrix, influencing tumour growth. Regions with varying immune cell presence may undergo swift mutations, exploring alternative metabolic pathways to evade immune clearance. By categorising tumour tissues into central and peripheral based on glucose tracer uptake differences, we aim to spatially classify tissues, providing a comprehensive understanding of metabolic changes in breast tumour regions.

Recent research on breast cancer has highlighted the variations in metabolism among different subtypes of the disease.^21^, ^22^ These metabolic differences have been shown to play a crucial role in tumour growth and progression, and understanding them is essential for developing effective treatments for breast cancer. ERs herein play a central role in metabolic regulation by interacting with various cellular key regulators, including hypoxia‐inducible factor, Ras/Raf/MAPK, PI3K/Akt/mTOR, p53 and c‐MYC.^23^

While HR status and HER2 status are commonly used to guide therapy and treatment, the documentation on how to make effective treatment choices based on tumour subtypes is currently limited.^24^ Even though pathological markers (ER, PR and HER2) can be used to differentiate between LumA and LumB breast cancers, and these markers are routinely used clinically to stratify patients for prediction and treatment selection, a pre‐clinical approach to differentiate between these two types and for subsequent treatment selection is lacking. Therefore, in an explorative approach we collected central and peripheral tumour tissues from LumA and LumB patients for metabolomic and transcriptomic comparison with glucose metabolism assessed employing positron emission tomography/magnetic resonance imaging (PET/MR), which facilitated our understanding of the biological heterogeneity of these two subtypes. This was done in an interdisciplinary research approach to link in vivo imaging with ex vivo molecular diagnostics, including advanced omics techniques to decipher tumour heterogeneity in human breast carcinoma.

## 2 METHODS

### 2.1 Ethical background

This study was approved by the ethics committee, Faculty of Medicine, University of Tübingen, Germany (516/2016BO1, study number GK‐PET/MR Tü018). All patients gave the written informed consent according the declaration of Helsinki. Sampling did not influence patient treatment. All data were pseudonymised according to European Data Protection Regulations and German law.

### 2.2 Collection and storage of patient specimens

All female patients with a first primary diagnosis of invasive breast cancer underwent surgery to remove the tumour. Tumour samples were collected from patients undergoing surgery at the Women's Hospital, University Hospital Tübingen, Germany. Before surgery, MRI and PET/MR images were reviewed in a joint session with the designated study radiologist (H. P.) and study pathologist (A. S.). Areas for sampling the fresh tissue were agreed on, with a preference for high‐uptake regions in the tumour periphery and a representative area in the tumour centre. Optimal sampling regions were documented in a standardised graph including sagittal and horizontal views of the breast. In the operating room, the resection specimen was completely put in an ice box and brought immediately to the pathology laboratory. The pathologist rapidly oriented the specimen according to the sampling map and performed the differential inking of the specimen according to four directions (ventral, dorsal, cranial and caudal). Serial sectioning was performed in 5–8 mm intervals along the mammilla–peripheral longitudinal axis of the segmentectomy or in sagittal silences of the mastectomy. The slices were laid out on in cold plate at −20°, which was covered by a single layer of cellulose (to prevent tissue from sticking to the metal). Regions of the tumour corresponding to the regions of interest were identified. Samples were taken with a sterile 3 mm single use punch biopsy from the centre and periphery. If tumours were too small for punching, the whole tumour was frozen. The tissue cores were placed in cryo‐ tubes, snap frozen and then stored at −80°C until analysis. Areas immediately surrounding the collection site were formalin fixed and embedded in paraffin blocks for further analysis by light microscopy, immunohistochemistry (IHC) and gene expression profiling by Nanostring PAM50. Patient information was collected, including standard demographics, cancer histology and the extent of peritoneal disease. Samples and data were pseudonymised. Of note, no tumour centre tissue with necrotic parts was used for this study.

### 2.3 Clinical histopathology

Samples were fixed in 10% neutral formalin for 24 h. Nodal and tumour samples were obtained by routine pathological techniques, such as haematoxylin and eosin (H&E) staining. Experienced pathologists evaluated all histopathological features, such as tumour size, histopathological type and grade. Histopathologic types were classified according to the 2012 WHO classifications. Histologic grades were assigned according to Elston and Ellis.^25^

The characterisation of HR and HER2 status was conducted on biopsy specimens prior to any treatment. The ASCO/CAP guidelines available during diagnosis were used to determine HER2 status.^26^ HER2‐low tumours were identified by IHC as 1+ or IHC 2+/ISH non‐amplified, while HER2‐0 was defined as IHC 0. The HR status was determined based on the currently available IHC data: tumours were classified as luminal‐like if the ER and/or PR levels were ≥1% or triple‐negative breast cancer if the ER and PR levels were both <1%. Additionally, the tumours were assigned a histologic grade according to the Nottingham histologic scoring system.^25^

All patients were classified into molecular subtypes based on Nanostring PAM 50 gene expression data performed on tumour RNA of the formalin fixed and paraffin embedded resection specimen. The data were confirmed by correlation with IHC for ER and PR (positive when > 1%) and Ki67 (>20% in LumB). In addition, HER2 analysis was performed, consisting of a combination of IHC and fluorescent in situ hybridisation (FISH) for *HER2* gene amplification if necessary (IHC unequivocally positive with score 3+, additional FISH performed when IHC score intermediate with score 2+).

### 2.4 TCGA databases analysis

The mRNA expression comparison with LumA and LumB was done by downloading respective data sets from The Cancer Genome Atlas (TCGA) database. The intrinsic subtypes of breast cancer, defined by differential expression of 50 genes called PAM50, including basal‐like, LumA, LumB, Her2 and normal‐like subtypes.^27^ The PAM50 signature of TCGA database was downloaded from the cancer immunome atlas (https://tcia.at/). The basal‐like (*n* = 218), Her2 (*n* = 131), LumA (*n* = 302), LumB (*n* = 267) and normal‐like (*n* = 126) molecular subtypes were compared. Principal component analysis (PCA) was applied to visualise breast cancer subtypes utilising the ‘limma’ and ‘ggplot’ R language packages. The survival analysis was calculated through the Kaplan–Meier curve. The gene set variation analysis (GSVA) package was used for single‐sample gene set enrichment analysis (ssGSEA) analysis to obtain a Hallmark gene set score. The MutationalPatterns R package was used to analyse mutational signatures in the targeted sequencing data, providing a diverse set of tools for assessing transcriptional and replicative strand bias, genomic distribution and association with genomic features.

### 2.5 PET/MR imaging

Our simultaneous PET/MR protocol included (i) a dedicated scan of the breast which was started 60 min after i.v. injection of the radiolabelled glucose analogue 2‐deoxy‐2‐[fluorine‐18]fluoro‐D‐glucose ([^18^F]FDG) (258 ± 4 MBq) (20 min PET emission recording) which was followed by a (ii) whole‐body PET/MR scan for oncological staging (4 min PET emission recording per bed position). PET emission data were reconstructed using an ordered subsets expectation‐maximization three‐dimensional (OSEM3D) algorithm, applying an MR‐based attenuation map from segmented MR images provided by the vendor. Additionally, an atlas‐based bone estimation was performed for attenuation correction, resulting in a maximal achievable resolution of 4.3 mm.^28^ Dedicated breast MR acquired a 4‐channel breast MRI coil (Siemens Healthineers, Erlangen, Germany) and dedicated MRI sequences (short time inversion recovery [STIR]), DW echo planar imaging (EPI; TR 11 000 ms, TE 76 ms, pixel size 1.8 × 1.8 mm^2^ in plane, 4 mm slice thickness, *b*‐values 50, 800 s/mm^2^), contrast enhanced (CE) dynamic imaging (T1 fast low‐angle shot [FLASH] 3D) and post contrast agent T1 FLASH 3D fat saturated (TR 8.75 ms, TE 4.21 ms, voxel size 0.9 × 0.9 × 0.9 mm^3^) were used. ADC maps were calculated from the diffusion‐weighted (DW) echo planar imaging (EPI).

For whole body PET/MR examination the following MR settings were performed: a transversal and coronal T2‐weighted turbo spin echo sequence, a coronal whole body STIR sequence in free breathing, whole body diffusion weighted imaging, whole body T1‐weighted volumetric interpolated breath‐hold examination sequence after intravenous injection of 0.1 mmol/kg gadolinium‐based MRI contrast media (Gadovist®; Bayer Vital GmbH, Leverkusen, Germany), a fluid attenuated inversion recovery sequence of the head as well as a contrast‐enhanced T1‐weighted 3‐D magnetisation prepared rapid gradient echo sequence of the head.

The injected dose of [^18^F]FDG patients received was adjusted to the patient body weight (average: 2.5 ± 0.60 MBq/kg). Data were acquired on a state‐of‐the‐art PET/MR scanner (Biograph mMR; Siemens Healthineers, Erlangen, Germany).

### 2.6 RNA isolation and gene expression assay

H&E‐stained sections of formalin‐fixed paraffin‐embedded (FFPE) tumour tissues were examined, and regions comprising representative invasive breast carcinoma were delineated on each slide. The tumour centre and periphery were defined using the outlined regions, and 10 μm thick sections were cut and macro dissected to eliminate surrounding normal tissue beyond the delineated area.

According to the manufacturer's instructions, RNA was extracted from macro‐dissected 5 μm paraffin sections using the Maxwell® RSC RNA FFPE Kit and the Maxwell® RSC Instrument (Promega, Madison, WI, USA). The RNA samples were evaluated for yield and purity using a NanoDrop 2000 Spectrophotometer (Thermo Fisher Scientific, Waltham, MA, USA). RNA quality and DV200 values were assessed using an Agilent Fragment Analyzer Instrument for quality control (QC) purposes. Samples that did not pass the analysis or QC were removed, resulting in a final set of nine LumA and 15 LumB tumour central and peripheral tissues that met the quality standards established by NanoString.

The NanoString Breast Cancer 360 assay (BC360TM) was used to measure gene expression on a NanoString nCounter® SPRINT Profiler (NanoString Technologies Inc., Seattle, WA, USA), including 758 gene‐specific probe pairs of the BC360 targets, 18 housekeeping genes used for normalisation, nine exogenous positive control RNA targets, and eight exogeneous negative control sequences. The BC360TM gene expression panel was incubated overnight in a solution containing 50–250 ng of total RNA at 65°C to enable hybridisation.

### 2.7 Transcriptomics analysis

The NanoString ROSALIND platform was employed for nCounter analysis, and nCounts of mRNA transcripts were normalised using the geometric means of 18 housekeeping genes (*ABCF1, ACTB, G6PD, GUSB, MRPL19, NRDE2, OAZ1, POLR2A, PSMC4, PUM1, RPLR2A, SDHA, SF3A1, STK11IP, TBC1D10B, TBP, TFRC* and *UBB*). Housekeeping probes for normalisation are selected based on the geNorm algorithm implemented in the NormqPCR R library1. The PCA was used for visualising the clusters of LumA and LumB centre and periphery samples. The GSVA package was used for ssGSEA analysis to obtain a Hallmark gene set score. The average score of hallmark pathway GSVA score for each subtype was shown by balloon plot using ggpubr package. In addition, the GSEA,^29^ the statistical significance was defined as NES > 1, *p* < .05, and the overrepresentation of indicated gene ontology (GO) biology process (BP) gene sets in the ranked gene lists presented by the normalised enrichment score (NES). Out of the 758 gene‐specific covered by the BC360 panel, 454 genes aligned with the Hallmarks gene sets. Additionally, 527 genes were associated with metabolism‐related GO BP gene sets.

### 2.8 Tissue metabolite extraction for metabolomics analysis

The LumA centre (*n* = 10) and periphery (*n* = 10), LumB centre (*n* = 8) and periphery (*n* = 8) tumour tissues were cryogenically pulverised using liquid nitrogen with the Covaris tube (Covaris, Woburn, MA, USA) and then transferred into adaptive focused acoustics‐compatible glass tubes. A two‐phase metabolite extraction protocol was implemented to avoid lipid signal overlap in the spectra. For total lipid extraction, 300 μL of methanol and 1000 μL of tert‐butyl methyl ether were added to the Covaris tube using filter‐containing pipette tips and vortexed until a homogeneous solution was obtained. Each sample was subjected to a 5‐minute ultrasound extraction using the ultrasonicator (E220 Evolution instrument; Covaris). Two hundred and fifty microliters of ultrapure water was added to achieve two‐layer phase separation. The polar phase was separated into HPLC glass vials tubes, evaporated to dryness using a speedvac (Thermo Scientific Savant SPD, Waltham, MA, USA) and used for the subsequent metabolomics analysis.

### 2.9 Metabolomics analysis of polar metabolites by ^1^H‐NMR spectrometry

The dry metabolite pellets were re‐suspended in a deuterated phosphate buffer adjusted to a pH of 7.4, containing 1 mM 3‐(trimethylsilyl) propionic‐2,2,3,3‐d_4_ acid sodium salt as an internal standard (Sigma–Aldrich Chemie, Taufkirchen, Germany). The supernatants were pipetted to a 1.7 mm nuclear magnetic resonance (NMR) spectrometer‐compatible tube (Bruker Biospin, Rheinstetten, Germany) and measured with a triple resonance 1.7 mm room temperature probe. The NMR spectra of individual samples at 298 K were recorded on a 14.1 Tesla ultra shielded NMR spectrometer (600 MHz proton frequency, Avance III HD; Bruker BioSpin, Ettlingen, Germany). The polar sample spectra were recorded using 1D nuclear Overhauser effect spectroscopy (NOESY) and Carr‐Purcell‐Meiboom‐Gill (CPMG) experiments. The 90° excitation pulse P1, for NOESYs, ranged from 5.68 to 6.11 μs, and for CPMGs, the range from 5.68 to 6.05 μs. The relaxation delay (D1) was uniformly set at 4.0 s for both NOESYs and CPMGs. The acquisition time for each NOESY and CPMG scan was set to AQ = 2.73 s. The number of acquisitions (NS) varied for different samples; for NOESYs it was 64 scans per sample, resulting in 58 min 48 s per analysis while for CPMGs, the number of scans ranged from 512 to 4096 (corresponding a maximum duration of 7 h 47 min 19 s) depending on signal to noise ratio and laboratory estimates of the dilution factor of samples. Receiver gain value was set to 84.54 and time domain to 65 536 data points. The spectral preprocessing was performed using Bruker TopSpin 3.6.1, and the metabolite assignment and quantification were carried out using ChenomX NMR Suite 8.5 software and the CPMG spectra.

### 2.10 Chemometrics and statistics

Metabolite concentrations of each sample were exported into a data matrix. The probabilistic quotient normalisation approach was used to normalise the data for dilution effects, with a reference sample serving as the basis for normalisation. Univariate, multivariate and correlation analysis was performed using the MetaboAnalyst 5.0 online platform.^30^ The paired *t*‐test was used for statistical comparison as the breast cancer tissue was divided into central and peripheral tissues from the same patient. The volcano plot was used to analyse the metabolites present in LumA and LumB, with a significant value of (pair *t*‐test) raw *p* values < .05 and fold change (FC) cut‐off > 1.2. Orthogonal projections to latent structures discriminant analysis (oPLS‐DA) score plots were used to see how central samples clustered with peripheral samples. The PCA bi‐plot was further analysed to identify specific metabolite involvement in different regions. Deviation plots were used to display the log_2_ fold change (log_2_FC) of metabolite concentrations between central and peripheral regions. Descriptive statistics and correlations were calculated with R software (version 4.2.2.), GSEA (version 4.2.2) and Prism GraphPad (version 8.0). Comparative statistics were performed using paired *t*‐test, *t*‐test, one‐way analysis of variance for normally distributed data and non‐parametric tests for skewed data. To mitigate the impact of multiple testing, we employed the false discovery rate (FDR) correction method. FDR‐adjusted *p* values were used in place of nominal *p* values to account for the increased risk of false positives, the Benjamini–Hochberg procedure was applied for FDR correction. Significant differences were considered at *p* < .05*; *p* < .01**; *p* < .001***; *p* < .0001**** in pair *t*‐test. Significant differences were considered at FDR < .05 in multiple tests. Graphical abstract created with BioRender.com.

## 3 RESULTS

### 3.1 The consort diagram of samples collection

**FIGURE 1:**
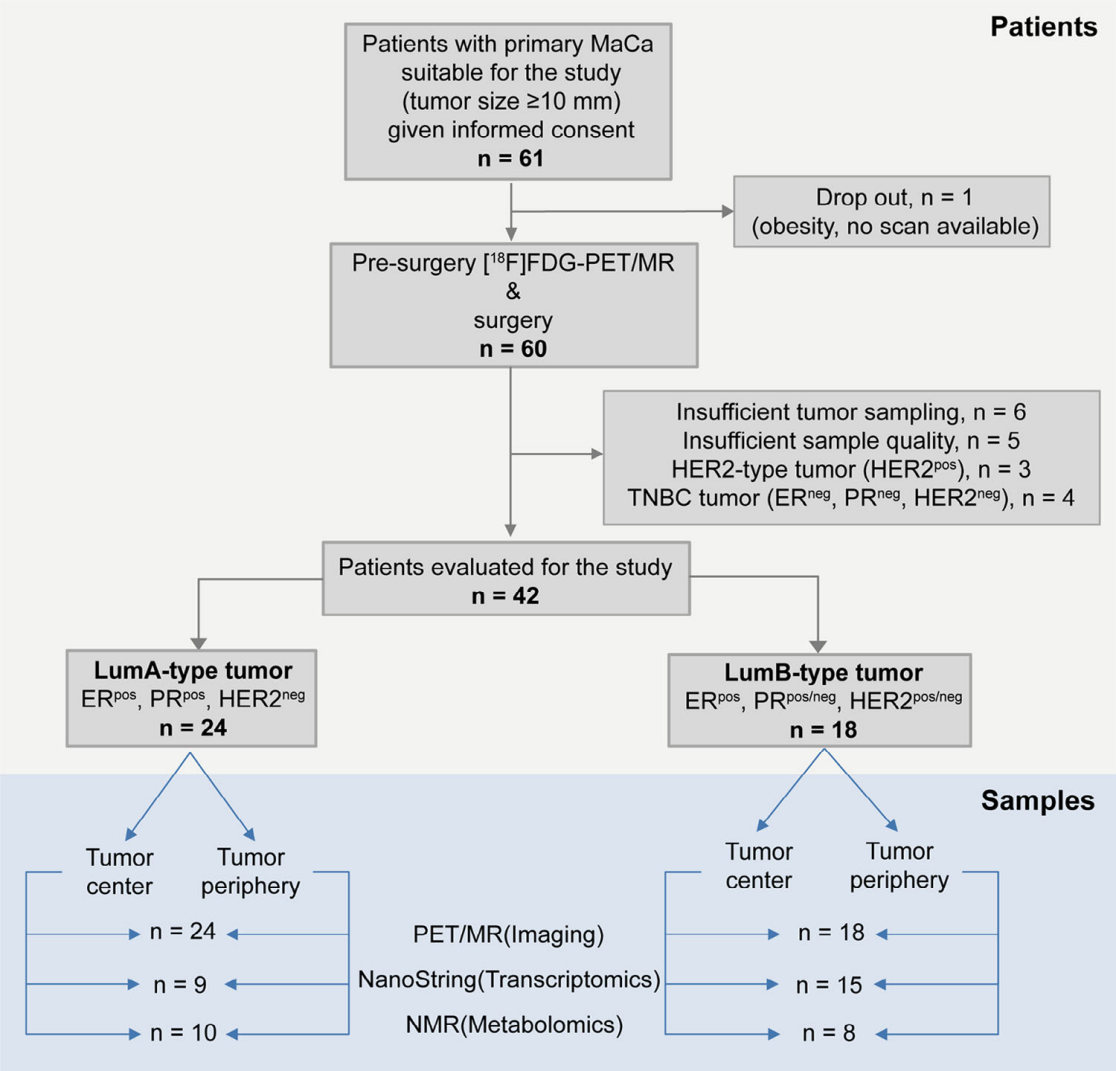
The Consort diagram of samples collection.

Figure 1 illustrates the samples and analysis types performed within this study. From a total of 61 patients that gave consent to participate in the study, 60 patients were examined by [^18^F]FDG‐PET/MR and out of those 42 deeply phenotype by transcriptomics and metabolomics analysis, respectively, focusing on LumA (*n* = 24) and LumB (*n* = 18).

### 3.2 Clinical information of patients

Table 1 illustrates the subtypes according to the IHC results, location of tumour and clinic‐pathological characteristics of the patients.

**TABLE 1:** Clinical‐pathological characteristics of the explorative samples’ cohort.

PET/MR (imaging)				
Subtype	Luminal A (LumA)		Luminal B (LumB)	
Location	Centre (*n* = 24)	Periphery (*n* = 24)	Centre (*n* = 18)	Periphery (*n* = 18)
Age (mean ± SD)	52.92 ± 11.36		58.44 ± 10.62			
BMI (kg/m^2^) (mean ± SD)	25.08 ± 4.31		29.02 ± 4.45			
Tumour size (mm) (mean ± SD)	24.08 ± 12.74		23.39 ± 7.79			
ER status	Positive (*n* = 24) 100%	Negative (*n* = 0) 0%	Positive (*n* = 18) 100%	Negative (*n* = 0) 0%		
PR status	Positive (*n* = 22) 92%	Negative (*n* = 2) 8%	Positive (*n* = 17) 94%	Negative (*n* = 1) 6%		
HER2 status	Positive (*n* = 0) 0%	Negative (*n* = 24) 100%	Positive (*n* = 5) 28%	Negative (*n* = 13) 72%		
Histology grade	G1 (*n* = 4) 17%	G2 (*n* = 16) 66%	G3 (*n* = 4) 17%	G1 (*n* = 0) 0%	G2 (*n* = 6) 33%	G3 (*n* = 12) 67%
Stage	I (*n* = 10) 42%	II (*n* = 13) 54%	III (*n* = 1) 4%	I (*n* = 8) 44%	II (*n* = 10) 56%	III (*n* = 0) 0%
T Stage	T1 (*n* = 11) 46%	T2 (*n* = 12) 50%	T3 (*n* = 1) 4%	T1 (*n* = 9) 50%	T2 (*n* = 9) 50%	T3 (*n* = 0) 0%
N Stage	N0 (*n* = 12) 50%	N1 (*n* = 11) 46%	N3 (*n* = 1) 4%	N0 (*n* = 13) 72%	N1 (*n* = 5) 28%	N3 (*n* = 0) 0%
M Stage	M0 (*n* = 24) 100%		M0 (*n* = 18) 100%	

NanoString (transcriptomics)				
Subtype	LumA		LumB	
Location	Centre (*n* = 9)	Periphery (*n* = 9)	Centre (*n* = 15)	Periphery (*n* = 15)
Age (mean ± SD)	54.89 ± 6.15		58.67 ± 9.90			
BMI (kg/m^2^) (mean ± SD)	27.15 ± 4.21		28.79 ± 3.72			
Tumour size (mm) (mean ± SD)	33.89 ± 13.96		23.87 ± 7.92			
ER status	Positive (*n* = 9) 100%	Negative (*n* = 0) 0%	Positive (*n* = 15) 100%	Negative (*n* = 0) 0%		
PR status	Positive (*n* = 9) 100%	Negative (*n* = 0) 0%	Positive (*n* = 14) 93%	Negative (*n* = 1) 7%		
HER2 status	Positive (*n* = 0) 0%	Negative (*n* = 9) 100%	Positive (*n* = 5) 33%	Negative (*n* = 10) 67%		
Histology grade	G1 (*n* = 1) 11%	G2 (*n* = 7) 78%	G3 (*n* = 1) 11%	G1 (*n* = 0) 0%	G2 (*n* = 4) 27%	G3 (*n* = 11) 73%
Stage	I (*n* = 0) 0%	II (*n* = 8) 89%	III (*n* = 1) 11%	I (*n* = 6) 40%	II (*n* = 9) 60%	III (*n* = 0) 0%
T Stage	T1 (*n* = 0) 0%	T2 (*n* = 8) 89%	T3 (*n* = 1) 11%	T1 (*n* = 7) 47%	T2 (*n* = 8) 53%	T3 (*n* = 0) 0%
N Stage	N0 (*n* = 1) 11%	N1 (*n* = 7) 78%	N3 (*n* = 1) 11%	N0 (*n* = 10) 67%	N1 (*n* = 5) 33%	N3 (*n* = 0) 0%
M Stage	M0 (*n* = 9) 100%		M0 (*n* = 15) 100%			

NMR (metabolomics)						
Subtype	LumA		LumB			
Location	Centre (*n* = 10)	Periphery (*n* = 10)	Centre (*n* = 8)	Periphery (*n* = 8)		
Age (mean ± SD)	56.80 ± 8.32		59.63 ± 9.94			
BMI (kg/m^2^) (mean ± SD)	27.84 ± 4.52		29.42 ± 3.61			
Tumour size (mm) (mean ± SD)	33.50 ± 13.22		25.63 ± 8.91			
ER status	Positive (*n* = 10) 100%	Negative (*n* = 0) 0%	Positive (*n* = 8) 100%	Negative (*n* = 0) 0%		
PR status	Positive (*n* = 10) 100%	Negative (*n* = 0) 0%	Positive (*n* = 7) 88%	Negative (*n* = 1) 12%		
HER2 status	Positive (*n* = 0) 0%	Negative (*n* = 10) 100%	Positive (*n* = 4) 50%	Negative (*n* = 4) 50%		
Histology grade	G1 (*n* = 1) 10%	G2 (*n* = 8) 80%	G3 (*n* = 1) 10%	G1 (*n* = 0) 0%	G2 (*n* = 2) 25%	G3 (*n* = 6) 75%
Stage	I (*n* = 0) 0%	II (*n* = 9) 90%	III (*n* = 1) 10%	I (*n* = 3) 38%	II (*n* = 5) 62%	III (*n* = 0) 0%
T Stage	T1 (*n* = 0) 0%	T2 (*n* = 9) 90%	T3 (*n* = 1) 10%	T1 (*n* = 3) 38%	T2 (*n* = 5) 62%	T3 (*n* = 0) 0%
N Stage	N0 (*n* = 2) 20%	N1 (*n* = 7) 70%	N3 (*n* = 1) 10%	N0 (*n* = 5) 62%	N1 (*n* = 3) 38%	N3 (*n* = 0) 0%
M Stage	M0 (*n* = 10) 100%		M0 (*n* = 8) 100%			

### 3.3 The genomic landscape of LumA and LumB in TCGA

**FIGURE 2:**
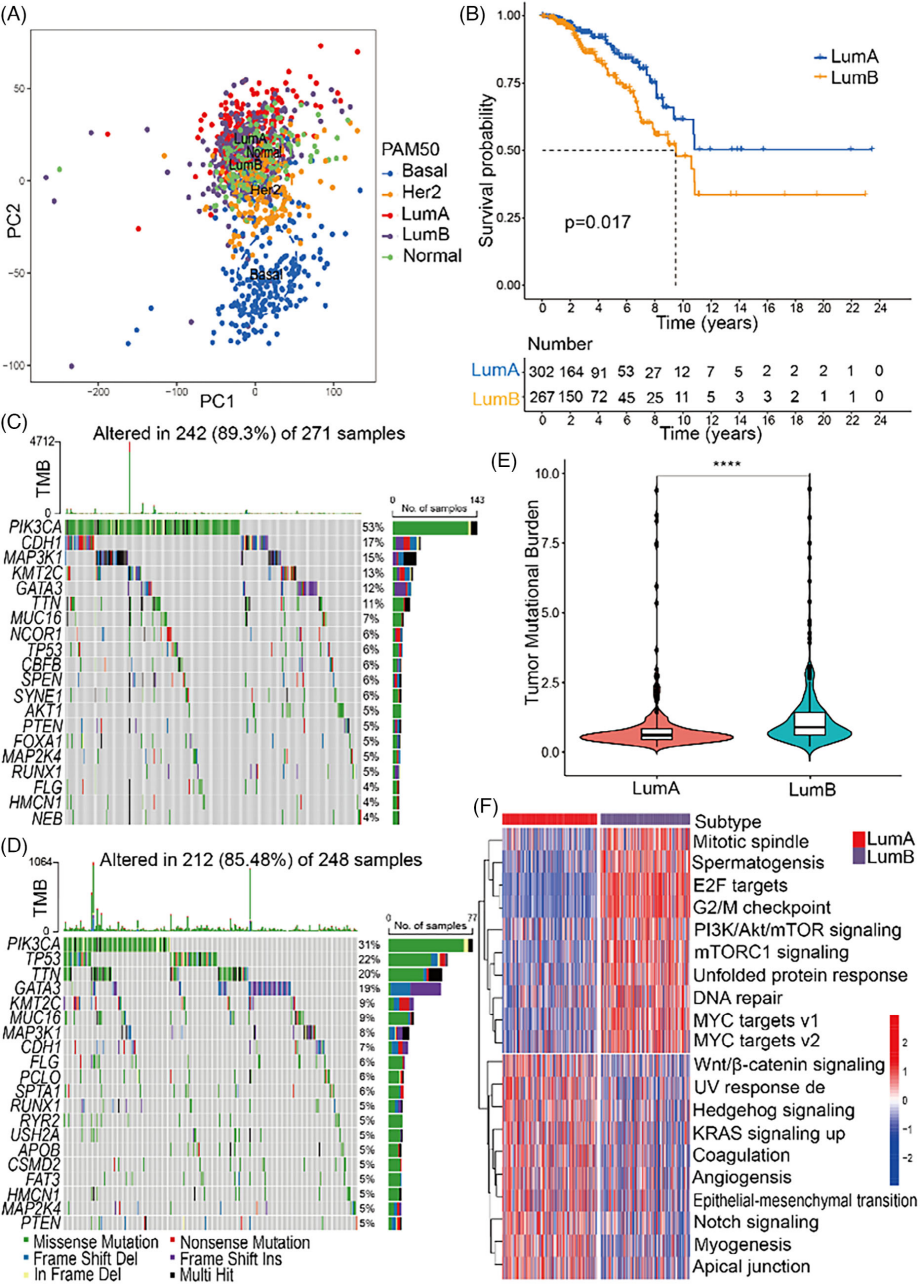
The genomic landscape of LumA and LumB based on public repository data of the TCGA database. (A) Principal component analysis (PCA) plot: the gene expression patterns of LumA and LumB tumours could not be distinguished clearly. (B) Kaplan–Meier curve: overall survival (OS) of patients with LumB tumours was lower than that of patients with LumA type. (C) Waterfall diagram: top 20 mutation genes in LumA, mutation of *PIK3CA* (53%), *MAP3K1* (15%), *KMT2C* (13%), *TP53* (6%), *PTEN* (5%), *AKT1* (5%) were related to cancer metabolism. (D) Waterfall diagram: top 20 mutation genes in LumB, mutation of *PIK3CA* (31%), *TP53* (22%), *GATA3* (19%), *KMT2C* (9%), *MAP3K1* (8%), *CDH1* (7%), *PCLO* (6%), *MAP2K4* (5%), *APOB* (5%), *PTEN* (5%), *RYR2* (5%) were related to cancer metabolism. (E) Tumour mutation burden: LumB is significantly higher than LumA (****p < .0001). (F) Top 20 differential hallmark pathways in LumA and LumB, LumB was more enriched on the pathways of tumour proliferation, and LumA was more enriched on the pathways of structural transformation (FDR < .05).

In order to better understand the metabolic pathway changes on the gene expression level, we analysed the TCGA database using the PAM50 classification algorithm for LumA and LumB. Although the two types could not be simply distinguished at the gene level, their differences pointed to metabolic and proliferative differences. The gene expression patterns of LumA and LumB could not be distinguished clearly by PCA (Figure 2A). The scree plot shows the percentage of explained variances by the first ten principal components (Figure S1A). However, there was a significant difference in between the overall survival rates of patients with LumA and LumB tumour types (Figure 2B). The results of somatic mutations in the 271 LumA and 248 LumB samples included in this study from the TCGA database showed that approximately 242 (89.3%) of the LumA and 212 (85.48%) of the LumB possessed somatic mutations. The top 20 most frequently mutated genes are illustrated in Figures 2C and D. The metabolism of tumour cells can be affected by mutations in some of genes, such as phosphatidylinositol‐4,5‐bisphosphate 3‐kinase catalytic subunit alpha (*PIK3CA*) (53%), mitogen‐activated protein kinase kinase kinase 1 (*MAP3K1*) (15%), lysine methyltransferase 2C (*KMT2C*) (13%), tumour protein P53 (*TP53*) (6%), phosphatase and tensin homolog (*PTEN*) (5%), serine/threonine kinase 1 (*AKT1*) (5%) in LumA, *PIK3CA* (31%), *TP53* (22%), GATA binding protein 3 (*GATA3*) (19%), *KMT2C* (9%), *MAP3K1* (8%), cadherin 1 (*CDH1*) (7%), piccolo presynaptic cytomatrix protein (*PCLO*) (6%), mitogen‐activated protein kinase kinase 4 (*MAP2K4*) (5%), apolipoprotein B (*APOB*) (5%), *PTEN* (5%), ryanodine receptor 2 (*RYR2*) (5%) in LumB. The tumour mutation burden was significantly higher in LumB than in LumA (Figure 2E). The GSVA score was applied to the transcriptome of breast cancer samples to screen the top 20 significant differences in hallmark pathways in LumA and LumB (Figure 2F) (FDR < .05). Herein, the tumour proliferation pathway, including G2M checkpoint and PI3K/Akt/TOR signalling pathways were highly expressed in LumB. In contrast, structural transformation pathway, including angiogenesis, myogenesis and epithelial–mesenchymal transition pathways were highly expressed in LumA. The top 20 differential expressed KEGG (Figure S2A), GO (Figure S2B) and REACTOME pathways (Figure S2C) between LumA and LumB were also illustrated (FDR < .05). Notably, the most significant differences in KEGG, GO BP and REACTOME pathways were consistently related to cell cycle pathways, with LumB exhibiting significantly higher enrichment than LumA.

### 3.4 PET/MR imaging and NanoString results in central and periphery tumour

Forty‐two patients with LumA and LumB breast cancer were recruited for PET/MR and metabolic changes in central and peripheral tumours areas were detected by measuring [^18^F]FDG uptake values. Detailed imagine data of the patients were presented in Table 2. The [^18^F]FDG SUV mean was significantly higher in the periphery than in the centre for LumA and LumB. The [^18^F]FDG SUV mean was higher in the centre and periphery of LumB than in LumA.

**TABLE 2:** Comparison of the central and periphery tumour by [^18^F]FDG PET/MR in LumA and LumB (paired *t*‐tests were used within regions of the same subtype and t‐tests between different subtypes).

Subtype	LumA (n = 24)			LumB (n = 18)		
Regions	Centre	Periphery	*p* Value	Centre	Periphery	*p* Value
Region‐of‐interest (ROI) (mm^2^)	11.80 ± 7.38	11.72 ± 5.21	.953	15.39 ± 7.33	13.85 ± 4.58	.166
Sub min (minimum enhancement within the ROI)	182.21 ± 107.75	219.33 ± 75.62	.055	175.61 ± 111.66	260.17 ± 60.84	.008^**^
Sub max (maximum enhancement within the ROI)	291.50 ± 118.37	347.71 ± 102.06	.011^*^	320.83 ± 103.15	380.89 ± 84.99	.015^*^
Kinetics initial (%)	203.63 ± 107.55	235.38 ± 94.07	.169	226.33 ± 137.25	283.00 ± 81.29	.028^*^
T2 mean (ms)	135.82 ± 47.72	122.67 ± 38.04	.126	155.96 ± 72.41	147.01 ± 65.05	.404
Apparent diffusion coefficient (ADC) mean (×10^−3^ mm^2^/s)	985.60 ± 285.18	928.69 ± 210.44	.379	1004.64 ± 427.58	882.56 ± 283.50	.220
Standardised uptake value (SUV) max	2.73 ± 1.52	2.15 ± 1.19	.0005***	4.71 ± 3.98	3.09 ± 1.75	.012^*^
Standardised uptake value (SUV) mean	2.65 ± 1.50	2.05 ± 1.11	.0005***	4.47 ± 3.67	2.94 ± 1.66	.009^**^

Regions	Centre			Periphery		
Subtypes	LumA	LumB	*p* Value	LumA	LumB	*p* Value
Region‐of‐interest (ROI) (mm^2^)	11.80 ± 7.38	15.39 ± 7.33	.122	11.72 ± 5.21	13.85 ± 4.58	.185
Sub min (minimum enhancement within the ROI)	182.21 ± 107.75	175.61 ± 111.66	.851	219.33 ± 75.62	260.17 ± 60.84	.074
Sub max (maximum enhancement within the ROI)	291.50 ± 118.37	320.83 ± 103.15	.417	347.71 ± 102.06	380.89 ± 84.99	.281
Kinetics initial (%)	203.63 ± 107.55	226.33 ± 137.25	.560	235.38 ± 94.07	283.00 ± 81.29	.101
T2 mean (ms)	135.82 ± 47.72	155.96 ± 72.41	.296	122.67 ± 38.04	147.01 ± 65.05	.145
Apparent diffusion coefficient (ADC) mean (×10^−3^ mm^2^/s)	985.60 ± 285.18	1004.64 ± 427.58	.866	928.69 ± 210.44	882.56 ± 283.50	.558
Standardised uptake value (SUV) max	2.73 ± 1.52	4.71 ± 3.98	.034^*^	2.15 ± 1.19	3.09 ± 1.75	.051
Standardised uptake value (SUV) mean	2.65 ± 1.50	4.47 ± 3.67	.038^*^	2.05 ± 1.11	2.94 ± 1.66	.049^*^

*p < .05.

**p < .01.

**FIGURE 3:**
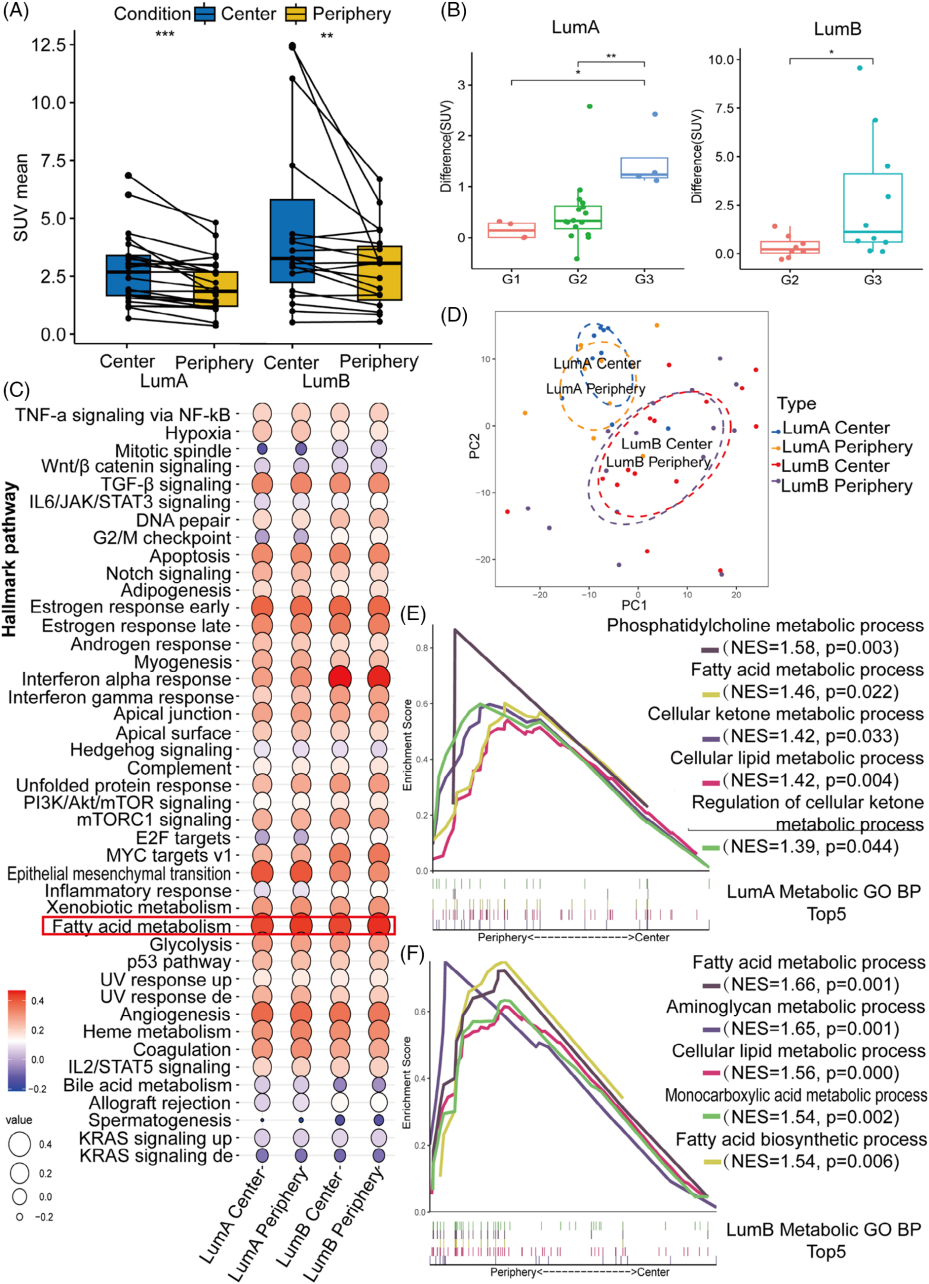
PET/MR imaging and NanoString in central and periphery tumour. (A) Box plot: the mean standardised uptake values (SUV) of [^18^F]FDG tracer was significantly higher in tumour centres of LumA (*n* = 24) and LumB (*n* = 18), and the SUV value of LumB was higher than LumA. (B) Box plot: the difference between the peripheral and central SUV means of LumA and LumB increased with tumour grade. (C) Balloon plot: the average gene set variation analysis (GSVA) score of central and peripheral of LumA and LumB. The score in fatty acid metabolism pathway were higher than glycolysis pathway. (D) Principal component analysis (PCA) plot: gene expression profiles in both the tumour centre and periphery were indistinguishable, but was distinct between LumA and LumB subtypes. (E) Gene set enrichment analysis (GSEA) analysis: the top five metabolic gene ontology (GO) biological process (BP) enrichment in tumour periphery compare with centre in LumA, all five processes were related to lipid metabolism‐related process (F) GSEA analysis: the top five metabolic pathway enrichment in tumour periphery compare with centre in LumB, which related to lipid and amino metabolism‐related processes (*p < .05, **p < .01, ***p < .001).

The box plot in Figure 3 illustrates significantly higher mean standardised uptake values (SUV) of [^18^F]FDG in tumour centres than in the periphery (Figure 3A), and the SUV mean of LumB was higher than that of LumA in both the centre and the periphery (Table 2). The difference between the peripheral and central SUV means of LumA and LumB both increased with tumour grade (Figure 3B). An overview of signature pathways based on the average GSVA scores of the LumA and LumB samples shows that the FA metabolism genome scores higher in these pathways (Figure 3C). According to the PCA plot, there was no noticeable distinction in gene expression between the tumour centre and periphery. However, there was a clear differentiation between the LumA and LumB subtypes (Figure 3D). The scree plot shows the percentage of explained variances by the first ten principal components (Figure S1B). GSEA showed that nine of the metabolism‐related GO bioprocesses (BPs) were significantly enriched in LumA centre (Table S1), and 26 of the metabolism‐related GO BPs were significantly enriched in LumB centre and 3 BPs were significantly enriched in periphery (Table S2). The enrichment scores of lipid metabolism‐related pathways in peripheral tumours were higher in LumB than in LumA (Figure S5). In LumA, the five metabolism‐related GO BP with the highest enrichment scores at the periphery compared with centre were phosphatidylcholine metabolic process (NES = 1.58, *p* = .002), FA metabolic process (NES = 1.46, *p* = .02), cellular ketone metabolic process (NES = 1.42, *p* = .03), cellular lipid metabolic process (NES = 1.42, *p* = .004) and regulation of cellular ketone metabolic process (NES = 1.40, *p* = .044) (Figure 2E). The five metabolism‐related GO BPs with the highest enrichment scores at the periphery compared with the centre in LumB were FA metabolic process (NES = 1.79, *p* = .000), aminoglycan metabolic process (NES = 1.75, *p* = .001), cellular lipid metabolic process (NES = 1.72, *p* = .000), monocarboxylic acid metabolic process (NES = 1.72, *p* = .000) and organic acid metabolic process (NES = 1.69, *p* = .003) (Figure 2F). The catabolic process of glucose was enriched in the central tumour of LumA (NES = −1.42, *p* = .08) and LumB (NES = −1.06, *p* = .36) (Figure S1), further validating the PET/MRI results that the tumour centre was dominated by glucose metabolism. The KEGG (Figure S3A) and RECOME (Figure S3B) pathways in LumA and LumB were also performed (NES > 1, *p* < .05).

### 3.5 Cell proliferation and lipid metabolism gene expression in tumour centre and periphery

**FIGURE 4:**
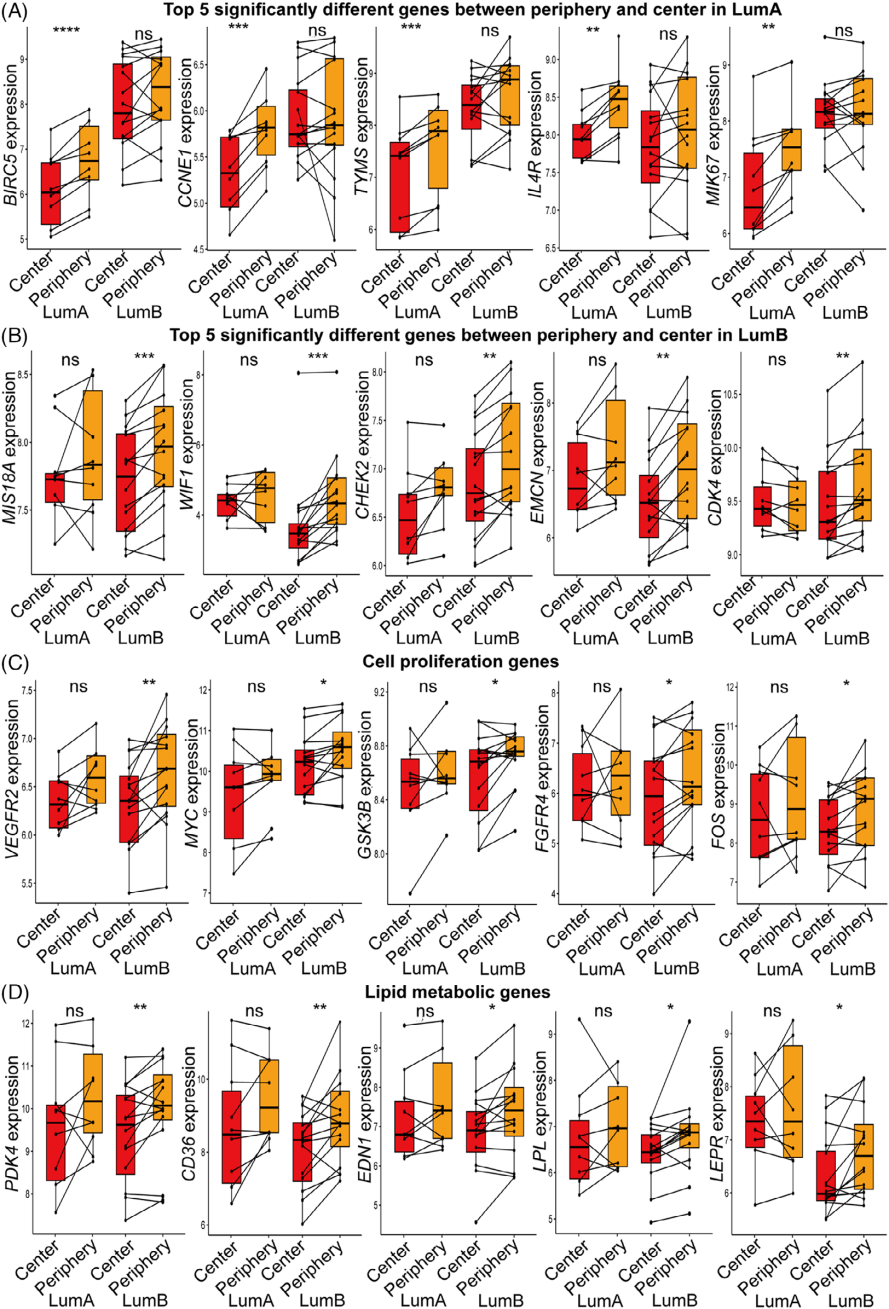
Differential genes in tumour centre and periphery tumour areas. (A) Top 5 significantly different genes between the tumour centre and periphery in LumA tumours, involved in cell proliferation, genomic instability and poor prognosis. (B) Top 5 significantly different genes between the tumour centre and periphery in LumB tumours, involved in tumour oncogenes, cell cycle, DNA damage response, Wnt signalling and tumour angiogenesis. (C) Differential cell proliferation genes: Cell proliferation related genes were significantly higher in LumB periphery, and also increased in the LumA periphery, but not statistically significant. (D) Lipid metabolism‐related genes: Lipid metabolism associated genes were significantly higher in peripheral of LumB tumours. The peripheral tumour of LumA tumours also showed an increase in these genes, but it was not significant (pair *t*‐test) (*p < .05, **p < .01, ***p < .001, ****p < .0001).

We identified 85 differentially expressed genes between the periphery and central tumour in LumA (Table S3), while only 58 genes in LumB were different (Table S4) (pair *t*‐test, *p* < .05). The box plots showed top five significant differences genes with significant differences between central and peripheral tumours in LumA, those were baculoviral IAP repeat containing 5 (*BIRC5*) (*t* = −7.59, *p* = .0001), Cyclin E1 (*CCNE1*) (*t* = −6.28, *p* = .0002), thymidylate synthetase (*TYMS*) (*t* = −5.08, *p* = .0009), interleukin 4 receptor (*IL4R*) (*t* = −4.93, *p* = .001) and marker of proliferation Ki‐67 (*MKI67*) (*t* = −4.70, *p* = .001) (Figure 4A). These genes are known to lead to increased proliferation of tumour cells, genomic instability and poor prognosis of patients.^31^, ^32^ Although these gene expressions were not statistically different between the centre and periphery of LumB, there was an observable trend indicating increased expression of these genes in the periphery. The top five significant different genes with significant differences between central and peripheral tumours in LumB were MIS18 binding protein 1A (*MIS18A*) (*t* = −4.56, *p* = .0004), WNT inhibitory factor 1 (*WIF1*) (*t* = −4.22, *p* = .0008), checkpoint kinase 2 (*CHEK2*) (*t* = −4.20, *p* = .0008), endomucin (*EMCN*) (*t* = −3.93, *p* = .001) and cyclin‐dependent kinase 4 (*CDK4*) (*t* = −3.88, *p* = .001) (Figure 4B). These genes act as tumour oncogenes, influence cell cycle control, DNA damage response, Wnt signalling and tumour angiogenesis. Cell proliferation‐related genes were significantly higher in peripheral tumour in LumB, including vascular endothelial growth factor receptor 2 (*VEGFR2*) (*t* = −3.16, *p* = .007), MYC proto‐oncogene (*MYC*) (*t* = −2.25, *p* = .04), glycogen synthase kinase 3 beta (*GSK3B*) (*t* = −2.6384, *p* = .01), fibroblast growth factor receptor 4 (*FGFR4*) (*t* = −2.44, *p* = .02) and FOS proto‐oncogene (*FOS*) (*t* = −2.51, *p* = .02). The expression of these genes also increased in the peripheral tumour of LumA, but the increase was not significant (Figure 4C). Lipid metabolism‐associated genes were significantly higher in peripheral tumours in LumB, like pyruvate dehydrogenase kinase 4 (*PDK4*) (*t* = −3.08, *p* = .008), CD36 molecule (*CD36*) (*t* = −3.09, *p* = .007), endothelin 1 (*EDN1*) (*t* = −2.48, *p* = .02), lipoprotein lipase (*LPL*) (*t* = −2.46, *p* = .02), leptin receptor (*LEPR*) (*t* = −2.66, *p* = .01). Their expression in the peripheral tumour of LumA also showed an increase, although it did not reach statistical significance threshold (Figure 4D).

### 3.6 Metabolic differences between tumour centre and periphery in different tumour subtypes

**FIGURE 5:**
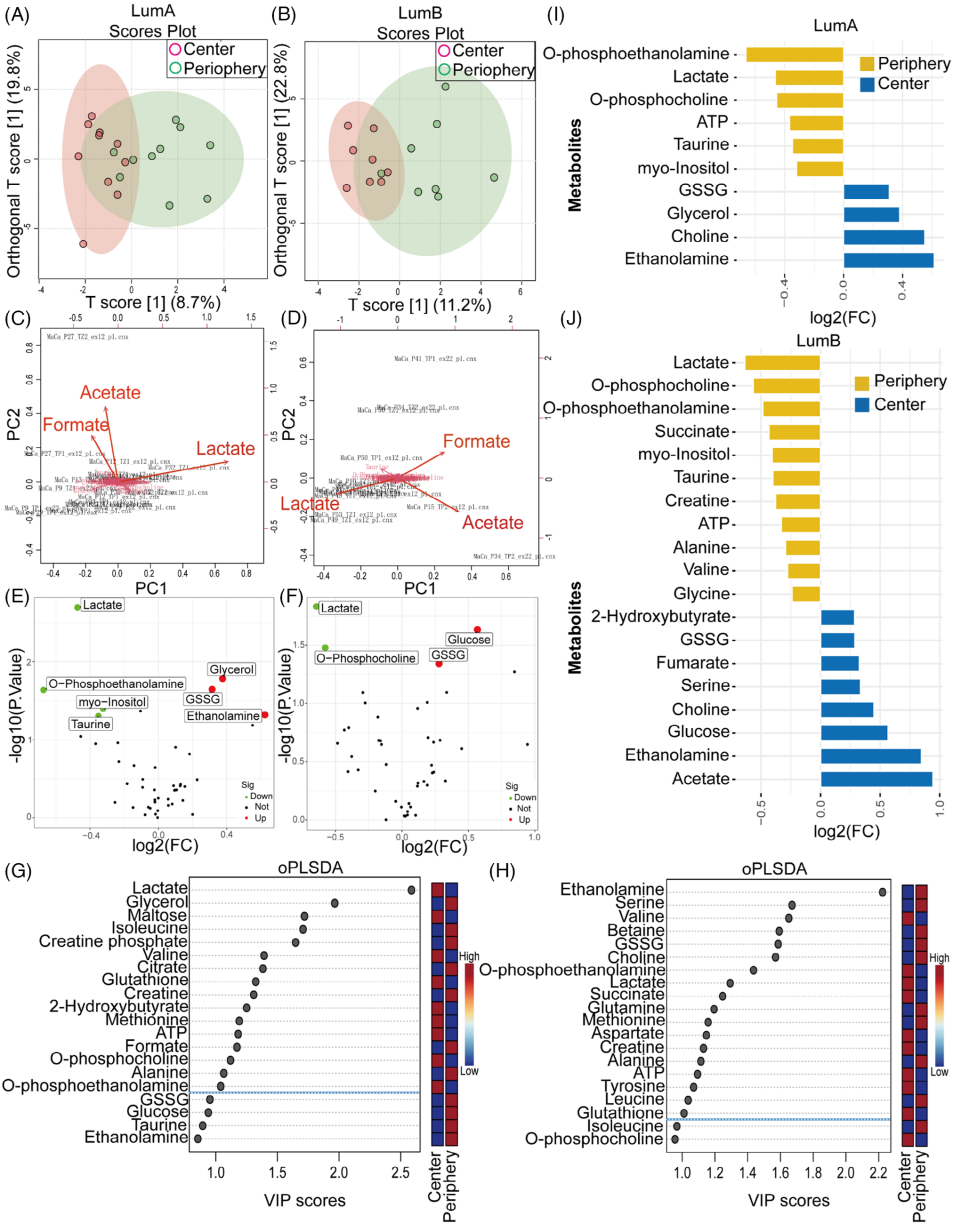
Metabolomics of the LumA and LumB tumours. (A and B) Orthogonal partial least‐squares discrimination analysis (oPLS‐DA) score plot: central and peripheral in LumA and LumB tumours illustrates centre comparable from periphery. (C and D) Principal component analysis (PCA) biplot illustrating the most important metabolites (acetate, formate and lactate) driving the separation of the principal components both in LumA and LumB tumours. (E and F) Volcano plot indicating statistically significant metabolites changes between the tumour centre and periphery: red plots mean up regular in periphery, and blue mean up regular in centre (*p* < .05, pair *t*‐test). (G and H) Partial least squares‐discriminant analysis (PLS‐DA) identifies 15 metabolites with variable importance in projection (VIP) scores > 1 in LumA tumours and 16 metabolites with VIP scores > 1 in LumB tumours. Blue dotted line indicates the PLS‐DA VIP score 1.0 threshold cut‐off. (I and J) Deviation diagram: differences in peripheral and tumour metabolites in LumA and LumB tumours, blue shows increase in peripheral tumours, yellow shows increase in central tumours (Fold change > 1.2). LumB tumours has more different metabolites than LumA tumours.

The oPLS‐DA score plot shows that the metabolic phenotype of LumA or LumB tumours is parallel in the centre and periphery (Figures 5A and B). Herein, acetate, formate and lactate were discriminated concerning other parameters in the PCA biplot of LumA and LumB (Figures 5C andD). The scree plot shows the percentage of explained variances by the first ten principal components of LumA and LumB (Figures S3A and B). In the volcano plot based on the FC > 1.2, *p* < .05 (pair *t*‐test), we identified that glycerol, glutathione disulfide (GSSG) and ethanolamine were increased, while lactate, O‐phosphoethanolamine, myo‐inositol and tyrosine were decreased in the periphery of LumA tumour (Figure 4E). In the periphery of LumB tumour, glucose and GSSG were up‐regulated, while lactate and O‐phosphoethanolamine were decreased (Figure 5F). To distinguish the most important metabolites between the centre and periphery, variable importance in projection (VIP) scores were used to screen for differential metabolites. Herein, lactate, glycerol, maltose, isoleucine, creatine phosphate, valine, citrate, glutathione, creatine, 2‐hydroxybutyrate, methionine, ATP, formate, O‐phosphocholine, alanine and O‐phosphoethanolamine showed a VIP score greater than 1 in LumA (Figure 5G). Ethanolamine, serine, valine, betaine, GSSG, choline, O‐phosphoethanolamine, lactate, succinate, glutamine, methionine, aspartate, creatine, alanine, ATP, tyrosine, leucine and glutathione PLS‐DA VIP score were more than 1 in LumB (Figure 5H). The deviation diagram showed peripheral concentrations compared with central concentrations for metabolites with FC > 1.2. A clear distinction could be observed between the peripheral and central metabolism in LumA and LumB. LumB has generally more differentiating metabolites than LumA. Glycerol concentration was higher only in peripheral tumours of LumA (Figure 5I). By contrast, creatine, alanine, valine and glycine concentrations were found to be elevated only in central tumours of LumB, while 2‐hydroxybutyrate, fumarate, serine, glucose and acetate concentrations were increased exclusively in peripheral tumours of LumB (Figure 5J). The heatmaps and correlation heatmaps of metabolites in the peripheral and central tumours of LumA and LumB are shown in Supplementary Figure S2.

### 3.7 Correlation of multi‐omics results

**FIGURE 6:**
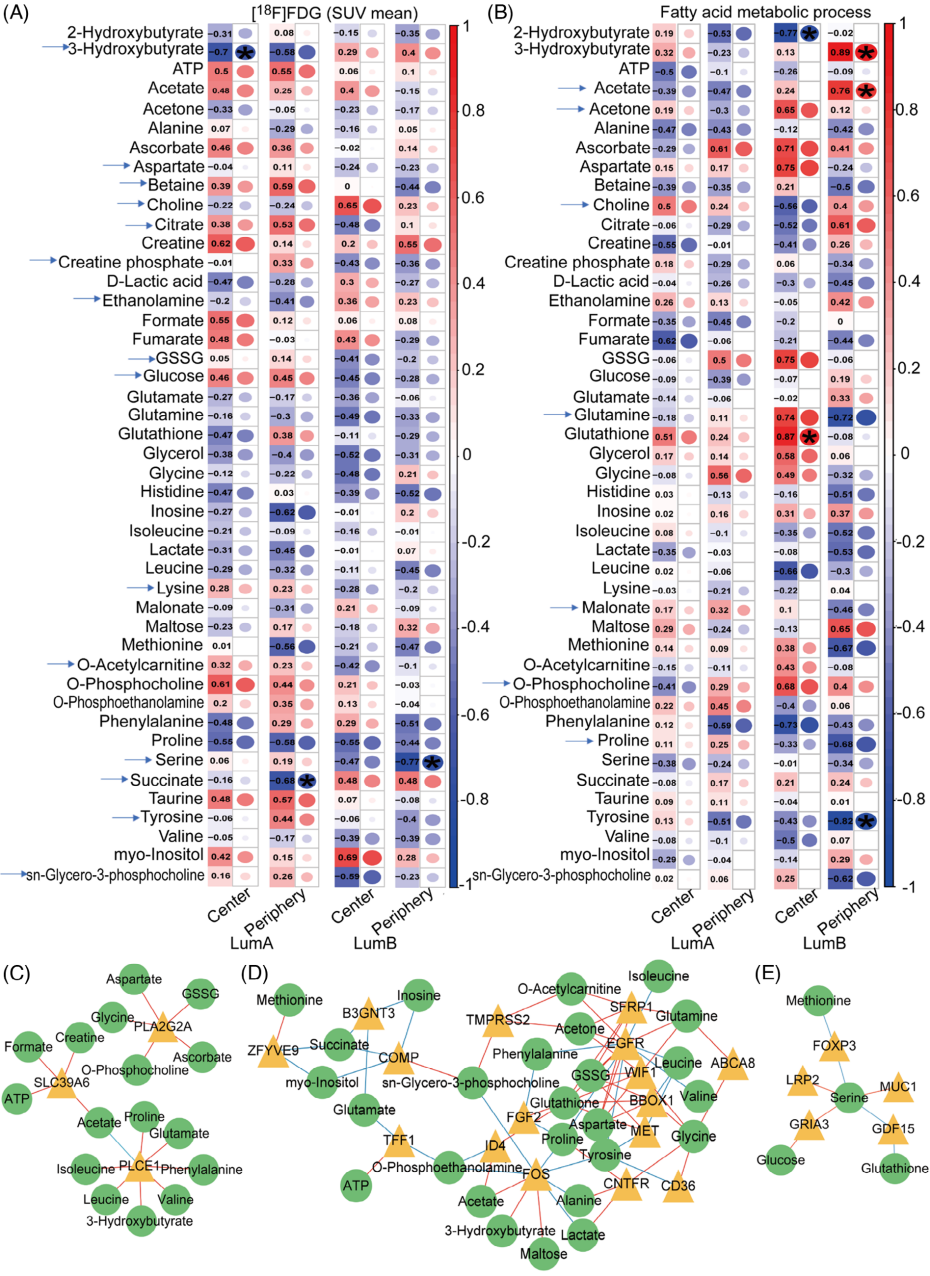
Correlation of multi‐omics results. (A) Correlation plot: the mean standardised uptake values (SUV) of [^18^F]FDG correlated with metabolites, red points show positive correlation, blue points show negative correlation (FDR < .05, *). (B) Correlation plot: the gene set variation analysis (GSVA) scores of fatty acid metabolic processes correlated with metabolites, red points show positive correlation, blue points show negative correlation (FDR < .05, *). Arrows in correlation plots indicate metabolites correlation in LumA and LumB are opposite. Correlation network: (C and D) lipid‐related statistically different genes associated with metabolites. Different genes are represented by yellow triangles, while metabolites are illustrated by green circles. The distance between the forms indicates the degree of closeness of the relationship. Red lines indicate positive correlation, while blue lines demonstrate negative correlation (*R* > .5, FDR < .05). There are more lipid metabolism genes correlated with metabolites in LumB than in LumA. (E) Serine‐related genes and their association with other one‐carbon unit metabolites (*R* > .45, FDR < .1).

The Spearman correlation method was employed to determine the correlation between the mean values of [^18^F]FDG uptake, GSVA scores and difference genes with metabolite concentrations. The correlation between the mean SUV of [^18^F]FDG and metabolites was different in LumA and LumB. The correlations between [^18^F]FDG tracer uptake and metabolites, including 3‐hydroxybutyrate, aspartate, betaine, choline, citrate, creatine phosphate, ethanolamine, GSSG, glucose, lysine, O‐acetylcarnitine, serine, succinate, taurine and sn‐glycero‐3‐phosphocholine were reverse in LumA and LumB (Figure 6A).

We next conducted correlation analysis of metabolites with GSVA scores from the GO FA metabolism process gene set, with the aim of identifying metabolites that may be associated with lipid metabolism. Herein, peripheral LumB FA metabolism showed positive correlations (*R* > .5) with 3‐hydroxybutyrate, acetate, citrate and maltose; and negative correlations (R< ‐.05) with betaine, glutamine, histidine, isoleucine, lactate, methionine, proline, tyrosine and sn‐glycero‐3‐phosphocholine (Figure 6B). Genes associated with lipid metabolism were selected from those genes statistically different at the periphery and centre of LumA and LumB.

In order to screen for core genes, correlation analysis was performed with metabolites. The correlations between peripheral and central differential genes and metabolites in LumA and LumB are presented in Tables S5 and S6. Although LumA shows a greater number of differentially expressed genes, only phospholipase C epsilon 1 (*PLCE1*), phospholipase A2 group IIA (*PLA2G2A*) and solute carrier family 39 member 6 (*SLC39A6*) were found to be associated with metabolites (*R* > .5, FDR < .05; Figure 6C and Table S5). *CD36*, MET proto‐oncogene (*MET*), epidermal growth factor receptor (*EGFR*), gamma‐butyrobetaine hydroxylase 1 (*BBOX1*), secreted frizzled related protein 1 (*SFRP1*), WNT inhibitory factor 1 (WIF1), ATP binding cassette subfamily A member 8 (*ABCA8*), fibroblast growth factor 2 (*FGF2*), inhibitor of DNA binding 4 (*ID4*), ciliary neurotrophic factor receptor (CNTFR), *FOS*, cartilage oligomeric matrix protein (*COMP*), zinc finger FYVE‐type containing 9 (*ZFYVE9*), UDP‐GlcNAc BetaGal beta‐1,3‐N‐acetylglucosaminyltransferase 3 (*B3GNT3*), transmembrane serine protease 2 (*TMPRSS2*), trefoil factor 1 (*TFF1*) were found to be associated with metabolites (*R* > .5, FDR < .05; Figure 6D and Table S6). *CD36* was positively correlated with *ID4* and *FOS*, which were positively correlated with acetate (FDR < .05; Figure S3). Serine is a crucial one‐carbon donor for proliferating cancer growth and survival and it was found higher in the periphery of LumB. We found one‐carbon related genes forkhead box P3 (*FOXP3*), mucin 1 (*MUC1*), growth differentiation factor 15 (*GDF15*), glutamate ionotropic receptor AMPA type subunit 3 (*GRIA3*), LDL receptor related protein 2 (*LRP2*) have correlations with serine (*R* > .45, *p* < .1) and some those genes have correlation with other one‐carbon metabolites (Figure 6E and Table S7).

### 3.8 Metabolic phenotypes in the tumour centre and periphery of LumA and LumB

**FIGURE 7:**
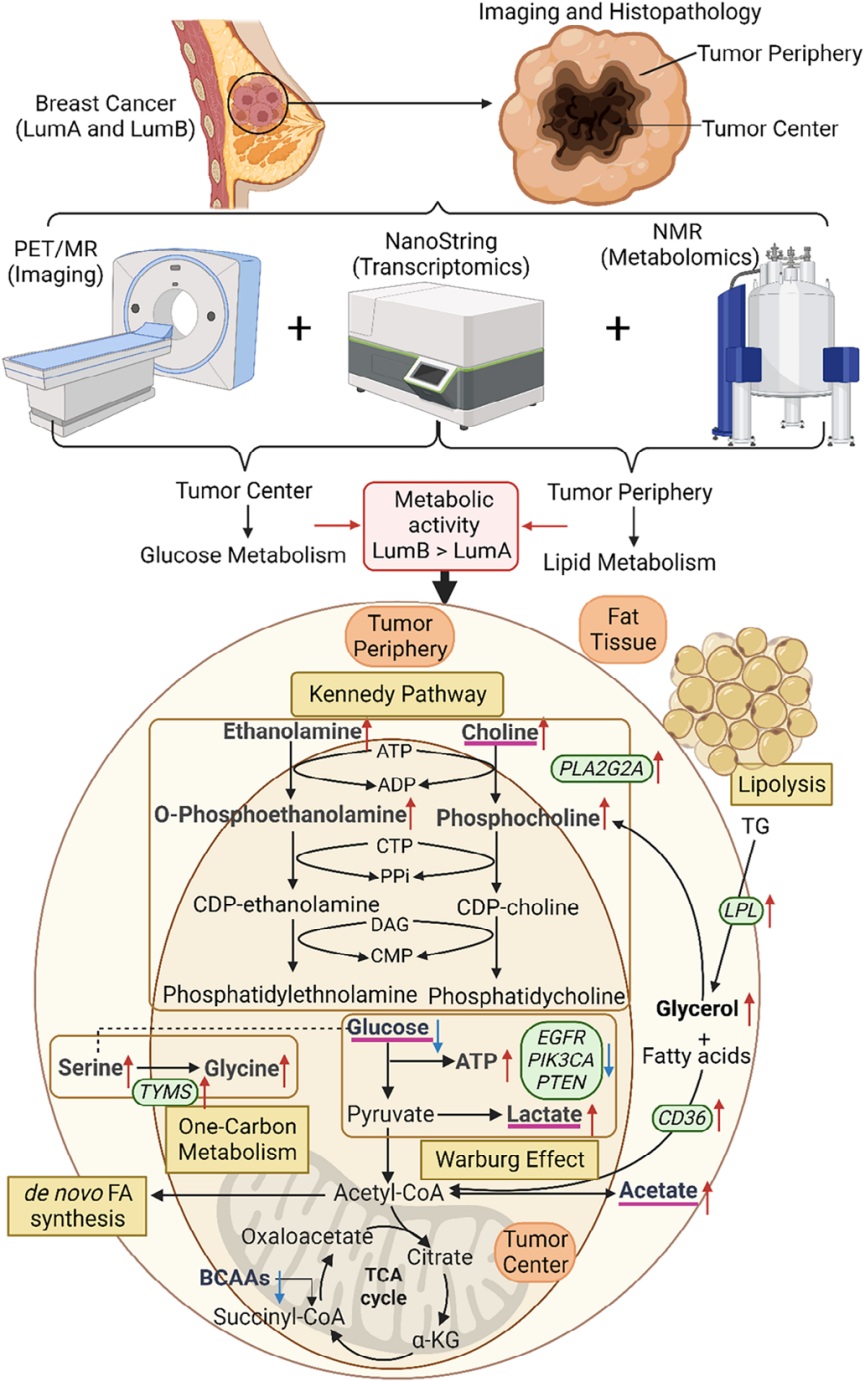
Graphical abstract to present metabolic heterogeneity in central and peripheral tumour areas of LumA and LumB. Red and blue line indicate positive and negative correlations and red and blue arrow means increase and decrease in central or peripheral areas. Metabolites highlighted in bold were detected within the NMR spectroscopy‐based metabolomics approach. Pink underlines indicate metabolites that are used as imaging tracers. Green circles represent significantly differentially expressed genes associated with metabolic pathways. α‐KG, α‐ketoglutarate; ATP, adenosine triphosphate; ADP, adenosine diphosphate; BCAAs, branched‐chain amino acids; CDP‐choline, cytidine diphosphocholine; CMP, cytidine monophosphate; CD36, Cluster of Differentiation 36 molecule; CDP‐ethanolamine, cytidine diphosphoethanolamine; CTP, phosphocholine cytidylyltransferases; DAG, diacylglycerol; EGFR, epidermal growth factor receptor; LPL, lipoprotein lipase; PLA2G2A, phospholipase A2 group IIA; PIK3CA, phosphatidylinositol‐4,5‐bisphosphate 3‐kinase catalytic subunit alpha; PPi, polyphosphoinositide; PDK4; pyruvate dehydrogenase kinase 4; TYMS, thymidylate synthase; TCA, tricarboxylic acid cycle.

We extensively analysed metabolic pathways and their association with gene expression levels to capture the metabolic heterogeneity between central and peripheral regions of breast tumours. In the metabolomics data set, the typical patterns of the Warburg effect were observed in the tumour centre and characterised by high lactate levels and low glucose levels. Cyclin‐dependent kinase 1 (*CDK1*), E2F transcription factor 1 (*E2F1*) and *PTEN*, all linked to the Warburg effect, exhibited significantly elevated expression levels in the periphery of LumA. Moreover, *PDK4*, *EGFR*, *PIK3CA* and Phosphoinositide‐3‐Kinase Regulatory Subunit 5 (*PIK3R5*), associated with the Warburg effect, displayed notable expression up‐regulation in the periphery of LumB (Figure S10A). Additionally, we identified the presence of the Kennedy pathway, known as cellular membrane growth metabolism, based on the high levels of O‐phosphoethanolamine and phosphocholine in the tumour centre, and high levels of ethanolamine and choline in the tumour periphery. The gene *PLA2G2A*, involved in the Kennedy pathway, exhibited a significant up‐regulation in expression in the periphery of LumA (Figure S10C). Furthermore, one‐carbon metabolism was identified with high glycine in tumour centre and high serine in tumour periphery. *TYMS*, a key gene in the one‐carbon metabolic process, also showed significant up‐regulation in expression in the periphery of LumA (Figure S10B). In addition, we observed increased levels of acetate and glycerol in the tumour periphery. Notably, the expression of *CD36*, a receptor with a pivotal role in lipid uptake and metabolism, and *LPL*, closely associated with triglyceride catabolism, exhibited significant up‐regulation in the periphery of LumB (Figure S10D). These findings collectively suggest that lipolysis is heightened in the peripheral mammary lipid tissue, contributing to energy regeneration within the tumours even with absence of glucose. For a visual summary of the metabolites and related gene expression in central and peripheral tumour regions, we produced a graphical abstract to present it (Figure 7).

Due to the potential impact of varying proportions of stromal and immune cells on metabolic heterogeneity within the TME, we employed the ESTIMATE method. This tool assesses tumour purity and evaluates the presence of stromal and immune cells based on gene expression data. Despite the significance of this analysis, our findings revealed that the ESTIMATE algorithm's stromal score (indicating the presence of stromal cells), immune fraction (indicating the presence of immune cells) and overall ESTIMATE score (reflecting overall tumour purity) did not exhibit notable differences between the central and peripheral regions of LumA and LumB tumours (Figure S11).

## 4 DISCUSSION

By employing multi‐omics techniques beyond classical singular parameter modalities, we can thoroughly comprehend of the evolving information landscape within a disease, covering its initial until the resulting functional consequences.^33^, ^34^, ^35^ In our study, we harnessed a multi‐omics approach to unveil the heterogeneity of tumours in primary human breast cancer and establish correlations with in vivo imaging tracer uptake studies. We confirmed the predominance of glucose metabolism in the centre of breast tumours, while lipid metabolism was more prominent in the periphery of the tumours. Furthermore, the overall metabolic activity based on glucose tracer uptake of LumB was higher than that of LumA. These results will aid the understanding of the metabolic heterogeneity in LumA and LumB subtypes, and correlation analysis of in vivo imaging and ex vivo molecular diagnostics could help the search for new candidates for metabolic tracers, enabling the differentiation between LumA and LumB subtypes using pre‐surgical tests, and could thus stratify patients for neoadjuvant therapy or optimised surgical protocols.

### 4.1 Metabolic heterogeneity in breast cancer

Increasing evidence shows that cancer cells exhibit heterogeneous metabolic requirements and preferences.^36^, ^37^ Understanding the emergence and evolution of metabolic heterogeneity in cancer is critical because it impacts our approach to utilise metabolic reprogramming for both cancer diagnostics and treatment. Herein, the metabolic adaptations of cancer are influenced by a range of intrinsic and extrinsic factors, which can significantly impact on tumour growth and progression, much like genomic or immune alterations. Intrinsic factors include genetic mutations or alterations in oncogenes or tumour suppressor genes, while extrinsic factors include changes in the TME, such as nutrient availability, oxygen levels and pH.^38^, ^39^

The major metabolic adaptation in cancer, known as the Warburg effect or aerobic glycolysis, provides cancer cells with the energy and building blocks needed for rapid proliferation.^40^ This study found that breast tumours exhibited a higher glucose uptake rate in their central region, accompanied by decreased glucose concentration and increased lactate. These findings thus suggest that cancer cells in the centre of the tumour rely heavily on glycolysis, a metabolic adaptation that may promote tumour progression, and they highlight the importance of understanding the metabolic adaptations of cancer cells in different regions of tumours.

In the periphery of tumours, we found different alterations: Metabolic differences between the centre and periphery of breast tumours may be related to the TME. Other researchers have observed that [^18^F]FDG uptake positively correlated with hypoxia and negatively correlated with cellular proliferation and tumour blood flow.^41^, ^42^ This is in line with our research that [^18^F]FDG uptake was higher in the central region, indicating more hypoxia and likely a lower proliferation. Furthermore, our research shows vascular endothelial growth factor receptor 2 (*VEGFR2*) was increased in the periphery, especially in LumB. CD34 Molecule (*CD34*) is a valuable vascular marker used in identifying and characterising endothelial cells, particularly those involved in angiogenesis and vascular biology, which was found to be negatively correlated with [^18^F]FDG uptake (Figure S11). This evidence may prove tumour vasculature may be enriched in the periphery. On the other hand, dysregulation in lipid metabolism is among the most prominent metabolic alterations to obtain alternative energy substrates, building blocks for biological membranes, and signalling molecules needed for proliferation, survival, invasion and metastasis.^43^, ^44^ Studies have consistently observed the aberrant choline phospholipid metabolism in breast cancer cells, highlighting a robust correlation with malignant progression.^45^ Furthermore, elevated de novo FA synthesis is imperative for the sustained proliferation of tumour cells, ensuring a constant supply of lipids, including phospholipids, for membrane synthesis.^46^ In our results, gene expression in the periphery is enriched in cellular lipid metabolic processes, phosphatidylcholine metabolic processes and FA metabolic processes.

Furthermore, in cancer, it is commonly observed that there is an increase in the levels of phosphoethanolamine, and an increase in phosphocholine, which is indicative of enhanced cell proliferation.^47^ The Kennedy pathway is the major route for forming of ethanolamine‐derived phospholipids, essential structural components of the cell membranes, known as cellular membrane growth metabolism.^48^ The accumulation can disturb the Kennedy pathway, impairing membrane function and signaling.^49^ This study found that higher ethanolamine and choline in the periphery and higher O‐phosphoethanolamine and phosphocholine in the centre, especially in LumB.

In one‐carbon metabolism, serine derived from glycolysis and exogenous uptake can be converted to glycine, providing the one‐carbon unit for one‐carbon metabolism.^50^ This metabolism can offer many intermediate metabolites as central precursors for synthesising of proteins, lipids and nucleic acids, forming a complex metabolic network for tumour progression.^51^ Serine is a major donor of one‐carbon units to the folate cycle through one‐carbon metabolism while producing glycine.^52^ Adenosine, guanosine and thymidylate are de novo synthesised in the folate cycle, which is necessary for the synthesis of nicotinamide adenine dinucleotide, nicotinamide adenine dinucleotide phosphate and ATP in mitochondria.^53^ The folate cycle is linked to the methionine cycle, which produces methyl groups that contribute to cellular biosynthesis and posttranslational modifications.^54^ The methionine cycle also provides precursors, such as cysteine for glutathione synthesis, which is essential for redox buffering.^55^ Thus, de novo serine metabolism may be required and adequate for tumour maintenance and promotion of oncogenesis.^9^ Our study indicates that serine increased in the periphery and glycine increased in the centre. Moreover, we found that some genes correlated with serine in breast cancer. The characteristic Treg transcription factors Foxp3 and Treg function are dramatically lost under various inflammatory conditions.^56^ Tregs with aberrant metabolism show increased serine metabolism but down‐regulation of *FOXP3*.^57^ In our results, *FOXP3* was negatively correlated with serine, therefore it is possible that serine can affect the TME, resulting in tumour proliferation and progression. *GDF15* has been reported to influence folate metabolism, a key component of one‐carbon metabolism,^58^ it was also negatively correlated with serine.

LumA and LumB peripheral genes were significantly enriched in lipid metabolism pathways. Acetate and *CD36* were significantly elevated in peripheral tumours of LumB in our results. While the reprogramming of glucose metabolism was the first recognised metabolic abnormality in tumour cells, there is increasing attention being given to the metabolic reprogramming of lipids in cancer,^59^, ^60^ especially for tumours that grow in a lipid‐rich environment, such as breast cancer. During cell proliferation, activated FA synthesis meets the demand for rapid membrane generation, while FAO provides the energy required for vigorous growth.^61^ During breast tumour invasion into surrounding normal tissues, tumour cells frequently encounter metabolic stress, such as hypoxia and nutrient deprivation, and therefore must absorb FAs and store lipids to generate energy for survival.^62^ Breast cancer cells demonstrate de novo FA synthesis, with increased expression of unsaturated FAs.^63^ Furthermore, FAO is more active in receptor‐positive breast cancers, similar to de novo FA synthesis.^22^ In breast cancer tissue, a significant proportion of the mesenchyme is occupied by cancer‐associated adipocytes, indicating that adipocytes play a substantial role in the TME.^64^ CD36, known as FA translocase, is also a crucial enzyme involved in the uptake of FAs, and evidence suggests that it contribute to breast cancer progression and up‐regulated in tumour cells and is responsible for the uptake of exogenous FAs into cell membranes.^65^

### 4.2 Crosstalk between metabolism and genes

Emerging evidence suggests that the activation of oncogenic pathways can up‐regulate specific metabolic pathways in cancer cells. In this study, we observed that cases of LumB breast cancer exhibited up‐regulation of oncogenic pathways that are closely associated with glycolysis, including the PI3K. The PI3K/AKT/mTOR pathway is frequently altered in luminal‐type tumours, with 40−50% of cases exhibiting mutations in pathway elements such as P*IK3CA*, *PIK3R1*, *PTEN* and *AKT1*.^66^ Among these genes, *PIK3CA* and *MAP3K1* are the most commonly mutated in this subtype.^67^ The TCGA database analysis we conducted produced identical results.

*BIRC5*, *CCNE1*, *CDK2*, *TYMS* and *MKI67* are the five most significant differential genes in LumA types. These genes significantly affect role in breast cancer development and progression, particularly in cell cycle regulation and proliferation. *BIRC5* is a human gene that encodes a protein critical in preventing apoptosis.^68^ *CCNE1* is a human gene that encodes a protein critical role in cell cycle regulation.^69^ The protein is a regulatory subunit of cyclin‐dependent kinase 2 (CDK2), a protein kinase that promotes cell cycle progression.^70^ *TYMS* is a human gene that encodes an enzyme that plays a key role in DNA synthesis by catalysing the conversion of deoxyuridine monophosphate to deoxythymidine monophosphate (dTMP).^71^ dTMP is a precursor of thymidine, an essential building block for DNA replication and repair. Ki‐67 (*MKI67*) is a protein that is commonly used as a marker to measure the proliferation rate of cells and determine the grade of certain tumours, detected through IHC staining.^72^

The top five differential genes in LumB tumours, ranked by significance, are *MIS18A*, *WIF1*, *CHEK2*, *EMCN* and *CDK4*. *MIS18A* is involved in chromosome segregation during cell division by maintaining the centromeres,^73^ whereas *WIF1* regulates the Wnt signalling pathway,^74^ which is essential for various cellular processes. *CHEK2* plays a crucial role in DNA damage response and cell cycle regulation, and mutations in this gene can increase cancer risk.^75^ *EMCN* regulates the permeability of blood vessels and may have implications for inflammation and tumour growth.^76^ CDK4 is an enzyme that works with cyclin D1 to promote cell cycle progression by activating Rb, and overexpression of CDK4 has been linked to certain types of breast cancer, as well as other cancers.^77^

Our results positively correlated *GRIA3*, *MUC1* and *LRP2* with serine in our results. GRIA3 is a receptor of glutamate involved in the glutamate signalling pathway, and glutamate is interconnected with one‐carbon metabolism through the generation of α‐ketoglutarate.^78^ MUC1 regulates carbon flux by directly modulating metabolic enzymes like PKM2,^79^ which may help accumulate precursor substances for one‐carbon metabolism.^80^, ^81^ LRP2 is involved in the internalisation and transport of folate‐binding proteins, such as folate receptor alpha (FOLR1), which is responsible for cellular uptake of folate, which is an essential component of one‐carbon metabolism.^82^

The key aspect of our study was dividing the tumours into peripheral and central sites and analysing the genetic variations. Although the initial differential genes identified are primarily involved in regulating the cell cycle and cell proliferation, they are not directly involved in metabolism. In general, the enrichment of lipid metabolism pathways in tumour periphery samples compared with tumour centre samples was observed for both LumA and LumB breast cancer.

### 4.3 Correlation of glucose uptake value and FA metabolism score with metabolites

Evidence suggests that ketosis, 3‐hydroxybutyrate (3‐HB), may significantly slow cancer progression in preclinical cancer models and patients.^83^, ^84^ A previous study has reported that exposing breast cancer cells to 3‐HB increased in glycolysis.^85^ According to our study, 3‐HB levels were higher in the centre of LumB breast tumours but lower in the periphery. Additionally, there was a positive correlation between 3‐HB levels and glucose uptake rate in LumB subtypes, while it was negatively correlated in LumA tumours. One possibility is that LumA and LumB tumours have distinct metabolic phenotypes, LumA typically has lower metabolic activity, while LumB have higher metabolic activity and rely more on glycolysis.^86^

Acetate and glycerol showed positively correlated with the GSVA score of the FA metabolism in LumB, especially in the periphery tumour. However, in LumA, this relationship was found to be the opposite. This could be attribute to the distinct molecular and metabolic profiles of LumB and LumA. Specifically, LumB tumours have been shown to have higher levels of lipid metabolism and exhibit a more aggressive phenotype than LumA. To satisfy the additional energy requirements, FA synthesis is a necessary metabolic change among all the reprogramming of metabolism, especially in the case of low cellular glucose uptake in which the regeneration of acetyl CoA from citrate is restrained.

*FOS* positively correlated with acetate and has been shown to regulate the expression of acetyl‐CoA synthetase (ACSS), an enzyme that catalyses the conversion of acetate to acetyl‐CoA and a key intermediate in lipid synthesis.^87^, ^88^ *ID4* positively correlated with acetate and was up‐regulated in the LumB periphery (Table S3). ID4 has been shown to regulate adipocyte differentiation involved in FA synthesis.^89^ ID4 is important for both mammary gland development and also for the etiologic of breast cancer.^90^, ^91^ *ID4* is overexpressed in a subset of breast cancer patients, marking patients with poor survival outcomes.^92^ According to our findings, ID4 can act as a transcriptional coactivator of the Kennedy pathway. Some studies have suggested that elevated levels of total choline and phosphocholine (PC) are consistently observed in aggressive forms of cancer.^93^, ^94^ Choline kinase‐α is frequently overexpressed in various types of cancers and is closely associated with tumour progression and invasiveness.^95^ Moreover, tumour proliferation involves changes in the composition of choline‐containing metabolites, characterised by elevated levels of choline and its phosphorylated metabolites in individuals with tumours.^94^ However, more research is needed to fully understand the relationship of the Kennedy pathway together with ID4 in breast cancer.

Towards the development of novel PET tracers, numerous publications focus on the potential of ^11^C‐acetate as a PET tracer in oncology.^96^, ^97^ A clinical trial has reported that combining PET examinations with FDG and ^11^C‐acetate provides added value in the diagnosis of hepatocellular carcinoma compared with single‐tracer imaging.^98^ The combination of ^11^C‐acetate and [^18^F]FDG may help to realise precise diagnosis avoiding false negative results and influencing the treatment methods depending on the final stage. The inverse correlation between acetate and serine in LumA and LumB subtypes holds promise as a nuclear medicine evidence to elucidate the distinction between these two subtypes in preclinical studies. Moreover, rapid proliferation is a distinguishing feature of peripheral tumour tissue, which can be attributed to the utilsation of adipose tissue or one‐carbon units. By leveraging metabolic markers,^99^ our objective is to identify specific metabolic characteristics linked to accelerated growth in peripheral tumours, offering valuable insights for guiding patients towards neoadjuvant therapy and optimising surgical procedures. For instance, patients exhibiting elevated lipid metabolism, may benefit from the complete removal of the adipose tissue surrounding the tumour and could potentially demonstrate heightened sensitivity to metabolic inhibitors that specifically target FA synthesis. Additionally, patients demonstrating heightened one‐carbon metabolism may benefit from using inhibitors targeting one‐carbon units.

### 4.4 Study limitations and future directions

Our investigation of the metabolomic and transcriptomic tissue heterogeneity within breast cancer is not without limitations. First of all, the cross‐sectional design of our study provides only a static snapshot of tumour heterogeneity at a specific moment, limiting our understanding of its dynamic evolution. Longitudinal studies, tracking changes in glucose uptake rates and molecular profiles over time, are therefore essential for a more nuanced comprehension of intra‐tumour heterogeneity. This is even more important as the division of tumours into central and peripheral regions was based on a single time point assessment of glucose uptake, overlooking potential temporal changes in spatial distribution and metabolic activity. That's why additional longitudinal assessments are imperative to capture the dynamic nature of tumour heterogeneity accurately.

Furthermore, our focus on transcriptomics and metabolomics analyses represents only a partial exploration of the full molecular omics landscape. The complexities of tumour biology extend to proteomics, epigenomics and immunopeptidomics, which were not addressed in our study. Integrating these additional layers of molecular analysis would help for a more comprehensive understanding of the intricate molecular variations within different tumour regions also towards their interaction with the immune system. Finally, novel technologies such as spatial metabolomics or ion mobility mass spectrometry would have helped to obtain a clearer picture upon metabolic pathways and in future studies we aim to integrate these techniques.

Moving forward, we envision leveraging the measurement of specific metabolic and gene markers associated with peripheral tumour growth and lipid metabolism. This strategic approach holds promise in guiding personalised decisions regarding neoadjuvant therapy and refining surgical protocols. By tailoring interventions based on the distinct metabolic signatures of individual tumours, we aspire to usher in a new era of precision medicine, optimising therapeutic outcomes and ultimately enhancing the quality of patient care in the realm of breast cancer management.

## 5 CONCLUSION

Our study provides a comprehensive framework for analysing the metabolic heterogeneity in human breast cancer by separating central and peripheral tumour tissues for imaging, transcriptomics and metabolomics studies, respectively. [^18^F]FDG uptake was higher in the tumour centre, indicating a prevalence of the Warburg effect, while genetic analysis revealed enrichment of lipid metabolism pathways in the tumour periphery, this defines the metabolic heterogeneity. Metabolomic differences in LumA and LumB subtypes further demonstrate heterogeneity in breast cancer. Through the identification of marker differential metabolites between subtypes, such as acetate, serine and choline, we aim to enhance the discovery of potential metabolic tracer candidates for preclinical subtype differentiation. Measurement of specific metabolic and gene markers related to peripheral tumour growth and lipid metabolism has the potential to guide patients toward neoadjuvant therapy and optimised surgical protocols.

## AUTHOR CONTRIBUTIONS

*Conception*: B. J. P., C. L. F., M. H., I. B., K. N. and C. T. *Design of the work*: B. J. P., C. L. F., M. H., I. B., K. N. and C. T. *Data acquisition*: Q. Y., A. K., H. P., T. C. S., A. K., G. B., A. F., I. B. and C. T. *Data analysis*: Q. Y. and C. T. *Interpretation of data*: Q. Y. and C. T. *Figure preparation*: Q. Y. *Manuscript draft*: Q. Y. and S. D. *Manuscript editing*: H. P., L. Z., B. G., A. S., A. D. H., B. J. P., C. L. F., M. H., I. B., K. N. and C. T. All authors have approved the submitted version.

## CONFLICT OF INTEREST STATEMENT

H. P. reports a restricted research fund from GE Healthcare. A. D. H. reports an institutional research grant from ExactScience. C. T. and B. J. P. report a research grant by Bruker BioSpin GmbH & Co. KG. Q. Y., S. D., T. C. S., A. K., L. Z., A. F., B. G., A. S., B. J. P., C. L. F., M. H., I. B. and K. N. declare that they have no competing interests.

## ETHICS STATEMENT AND CONSENT TO PARTICIPATE

Written informed consent was obtained from each patient prior to obtaining the sample to use for research purposes. All participants were given written consent by the declaration of Helsinki. Analyses of tumour samples was approved by the Ethics Committee, University of Tübingen, Germany (Ref. Nr. 516/2016BO1).

## Supporting information

Supporting Information

## Data Availability

The data presented are partially obtained by analysing already publicly available data. The datasets used and analysed during the current study are available from the corresponding author on reasonable request. ^1^H‐NMR spectra data and NanoString gene expression data are available upon request.
