# Supplementary material for: Image‐guided metabolomics and transcriptomics reveal tumour heterogeneity in luminal A and B human breast cancer beyond glucose tracer uptake
Source: Clin Transl Med. 2024 Feb 8;14(2):e1550. doi: 10.1002/ctm2.1550 (PMC10853679; doi:10.1002/ctm2.1550)
Supplement: Supplementary file 1 — Supporting Information [file CTM2-14-e1550-s001.docx]

**Image-guided metabolomics and transcriptomics reveal tumor heterogeneity in luminal A and B human breast cancer beyond glucose tracer uptake**

Qianlu Yang^1^, Sisi Deng^1,6^, Heike Preibsch^3^, Tim-Colin Schade^2^, André Koch^4^, Georgy Berezhnoy^1^, Laimdota Zizmare^1,6^, Anna Fischer^2^, Brigitte Gückel^3,6^, Annette Staebler^2^, Andreas D. Hartkopf^4^, Bernd J. Pichler^1,6,7^, Christian la Fougère^5,6,7^, Markus Hahn^4^, Irina Bonzheim^2,*^, Konstantin Nikolaou^3,6,7*^, Christoph Trautwein^1,6*^

^1^Werner Siemens Imaging Center, Department of Preclinical Imaging and Radiopharmacy, University Hospital Tuebingen, Germany

^2^Institute of Pathology and Neuropathology, University Hospital Tuebingen, Germany

^3^Department of Diagnostic and Interventional Radiology, University Hospital Tuebingen, Germany

^4^Department of Women’s Health, University Hospital Tuebingen, Germany

^5^Institute for Nuclear Medicine and Clinical Molecular Imaging, University Hospital Tuebingen, Germany.

^6^Cluster of Excellence iFIT (EXC 2180) “Image Guided and Functionally Instructed Tumor Therapies”, University of Tübingen, Tübingen, Germany

^7^German Cancer Research Center, German Cancer Consortium DKTK, Partner Site Tübingen, Tübingen, Germany

* Authors to whom correspondence should be addressed

[irina.bonzheim@med.uni-tuebingen.de](mailto:irina.bonzheim@med.uni-tuebingen.de); [konstantin.nikolaou@med.uni-tuebingen.de](mailto:konstantin.nikolaou@med.uni-tuebingen.de); [christoph.trautwein@med.uni-tuebingen.de](mailto:christoph.trautwein@med.uni-tuebingen.de)

**Supplement**

| 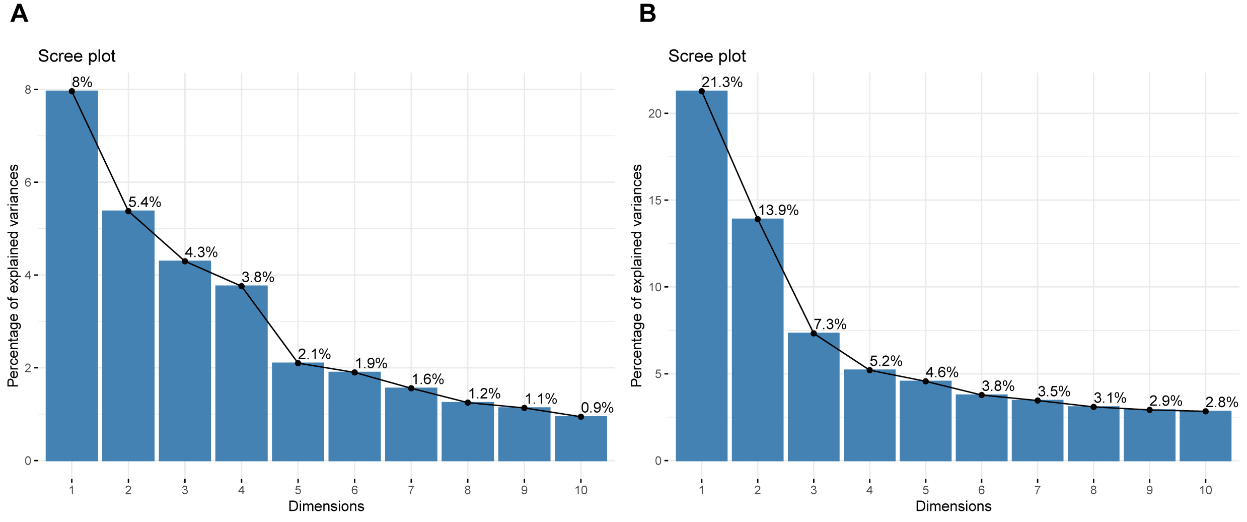 |
| --- |
| **Supplementary Figure 1. Scree plots of eigenvalues after principal component analysis (PCA)** A) The scree plot showing the percentage of explained variances by the first ten principal components of the TCGA PAM50 subtype. B) The scree plot showing the percentage of explained variances by the first ten principal components in the tumor center and periphery of LumA and LumB. |

| 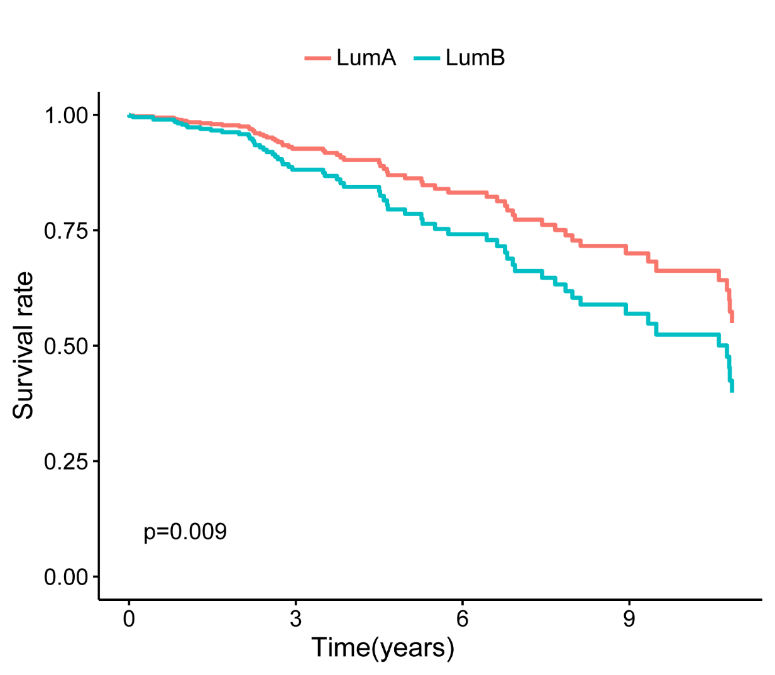 |
| --- |
| **Supplementary Figure 2.** **Kaplan-Meier curve of LumA and LumB.** Overall survival (OS) of patients with LumB classifies tumors was lower than that of patients with LumA type after age, stage, T, and N stage adjusted. |

| 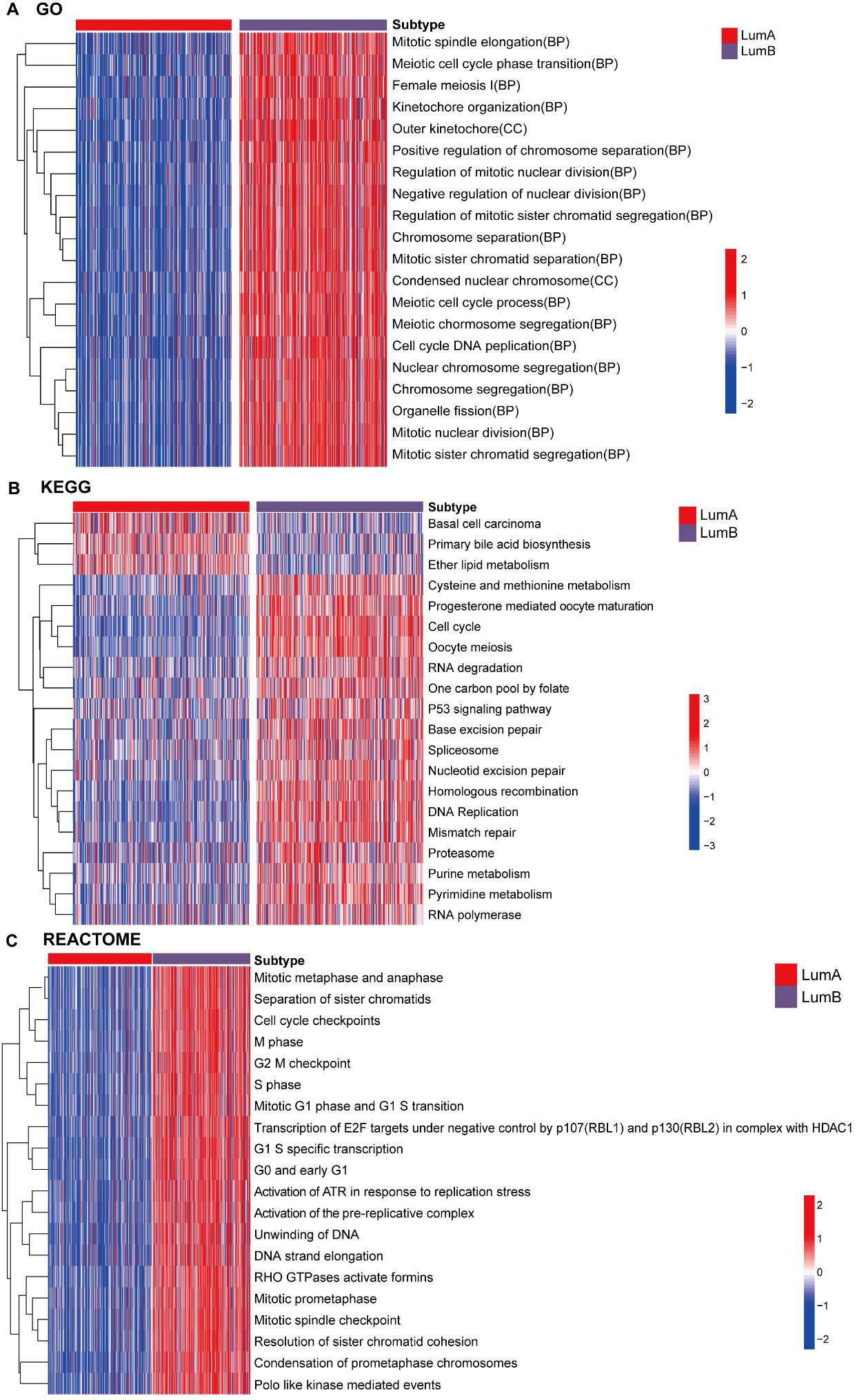 |
| --- |
| **Supplementary Figure 3. GO, KEGG, REACTOME pathway heatmap in LumA and LumB.** A) Top 20 differential enriched KEGG pathways in LumA and LumB (FDR<0.05). B) Top 20 differential enriched GO BP pathways in LumA and LumB (FDR<0.05). C) Top 20 differential enriched REACTOME pathways in LumA and LumB (FDR<0.05). The most significant differences in KEGG, GOPB and REACTOME pathways were all related to cell cycle pathways, with LumB significantly higher than LumA. |

**Supplementary Table 1. Metabolism-related gene sets enriched in phenotype periphery of LumA (NES>1, p<0.05)**

| Gene ontology biological process | NES | p-value |
| --- | --- | --- |
| Phosphatidylcholine metabolic process | 1.581 | 0.003 |
| Fatty acid metabolic process | 1.461 | 0.022 |
| Cellular ketone metabolic process | 1.424 | 0.033 |
| Cellular lipid metabolic process | 1.422 | 0.004 |
| Regulation of cellular ketone metabolic process | 1.404 | 0.044 |
| C21 steroid hormone metabolic process | 1.387 | 0.048 |
| Monocarboxylic acid metabolic process | 1.366 | 0.030 |
| Lipid metabolic process | 1.329 | 0.017 |

**Supplementary Table 2. Metabolism-related gene sets enriched in phenotype periphery and center of LumB (NES>1, p<0.05)**

| Gene ontology biological process | NES | p-value |
| --- | --- | --- |
| Fatty acid metabolic process | 1.659 | 0.001 |
| Aminoglycan metabolic process | 1.653 | 0.001 |
| Cellular lipid metabolic process | 1.562 | 0.000 |
| Monocarboxylic acid metabolic process | 1.543 | 0.002 |
| Fatty acid biosynthetic process | 1.541 | 0.006 |
| Polyol metabolic process | 1.535 | 0.009 |
| Organic acid metabolic process | 1.525 | 0.007 |
| Cellular carbohydrate metabolic process | 1.511 | 0.014 |
| Unsaturated fatty acid metabolic process | 1.506 | 0.015 |
| Unsaturated fatty acid biosynthetic process | 1.498 | 0.014 |
| Regulation of gluconeogenesis | 1.483 | 0.012 |
| Carbohydrate metabolic process | 1.482 | 0.025 |
| Icosanoid metabolic process | 1.473 | 0.023 |
| Aminoglycan catabolic process | 1.470 | 0.013 |
| Regulation of lipid metabolic process | 1.458 | 0.007 |
| Mucopolysaccharide metabolic process | 1.444 | 0.035 |
| Prostanoid metabolic process | 1.441 | 0.041 |
| Lipid metabolic process | 1.408 | 0.007 |
| Regulation of cellular ketone metabolic process | 1.408 | 0.049 |
| Alcohol metabolic process | 1.407 | 0.038 |
| Small molecule metabolic process | 1.394 | 0.011 |
| Long chain fatty acid metabolic process | 1.390 | 0.035 |
| ncRNA metabolic process | 1.378 | 0.046 |
| Carbohydrate derivative metabolic process | 1.322 | 0.047 |
| Positive regulation of phosphorus metabolic process | 1.284 | 0.031 |
| Positive regulation of protein metabolic process | 1.222 | 0.046 |
| Androgen metabolic process | -1.758 | 0.006 |
| Positive regulation of collagen metabolic process | -1.626 | 0.004 |
| Positive regulation of hormone metabolic process | -1.617 | 0.045 |

| 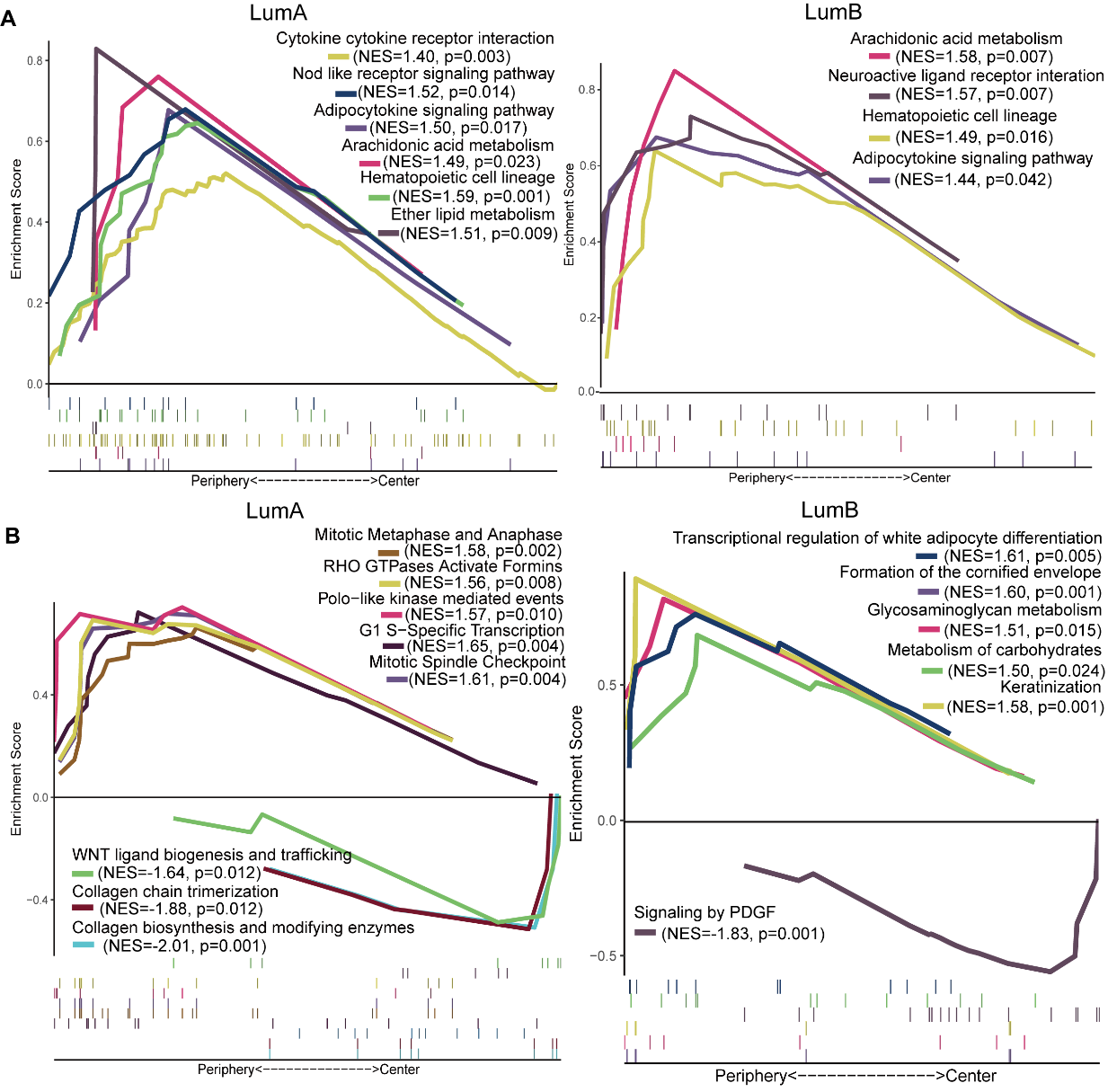 |
| --- |
| **Supplementary Figure 4. Results of KEGG and REACTOME from GSEA in the periphery and center of LumA and LumB.** A) The significantly enriched gene sets in KEGG in the periphery of LumA and LumB (no significant enriched gene set in KEGG for the center). B) The top 5 enriched gene sets in REACTOME for the periphery of LumA and LumB, with only three and one gene set enriched in the central of LumA and LumB, respectively. |

| 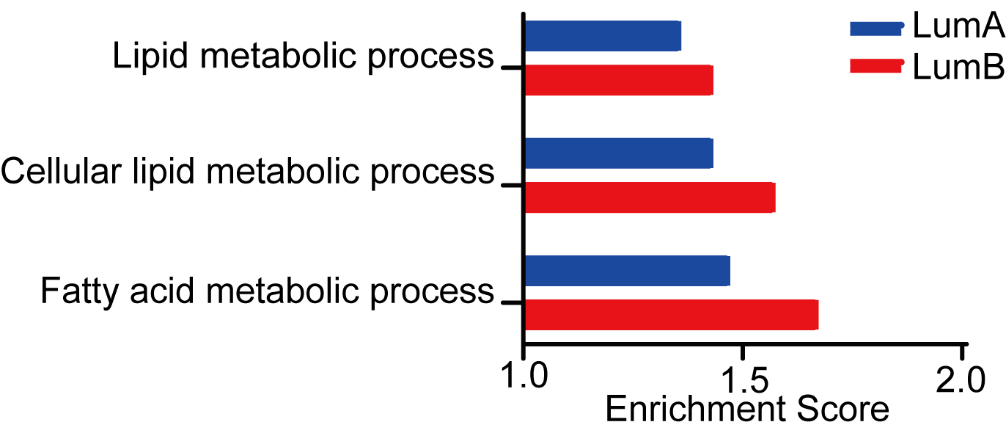 |
| --- |
| **Supplementary Figure 5.** Comparing enrichment scores of lipid metabolic processes in LumA and LumB, LumB is higher than LumA in all three processes. |

| 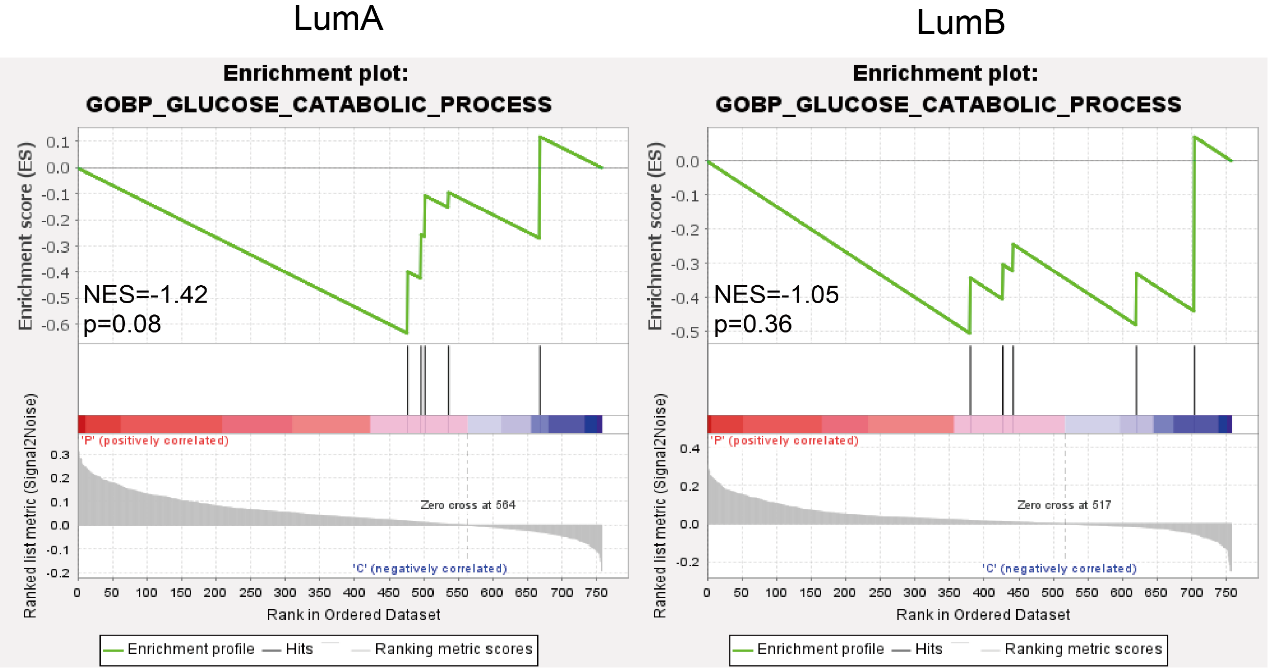 |
| --- |
| **Supplementary Figure 6. Glucose catabolic process enrichment in central tumor of LumA and LumB.** The glucose catabolic process was enriched in the tumor center of LumA and LumB, this result further validated the PET/MR results that the tumor center was dominated by glucose metabolism. |

**Supplementary Table 3. Differential genes in peripheral and central tumors in LumA (p<0.05) (pair t-test)**

| Gene | t-stat | *p*-value | Gene | t-stat | *p*-value |
| --- | --- | --- | --- | --- | --- |
| BIRC5 | -7.5852 | 6.39E-05 | RAD54L | -2.8959 | 0.02001 |
| CCNE1 | -6.2811 | 0.0002 | CCL2 | -2.8911 | 0.0202 |
| TYMS | -5.0764 | 0.0010 | CDC25A | -2.876 | 0.0206 |
| IL4R | -4.9321 | 0.0011 | KAT2B | -2.838 | 0.0219 |
| MKI67 | -4.7038 | 0.0015 | PLA2G2A | -2.7686 | 0.0243 |
| ANLN | -4.6376 | 0.0017 | PIK3R5 | -2.7477 | 0.0251 |
| CDKN2D | -4.5003 | 0.0020 | AURKB | -2.7388 | 0.0255 |
| IL6R | -4.479 | 0.0021 | NEIL1 | -2.7348 | 0.0257 |
| HELLS | -4.2637 | 0.0027 | TIE1 | -2.7345 | 0.0257 |
| PTEN | -4.1079 | 0.0034 | ROBO4 | -2.7042 | 0.0269 |
| PECAM1 | -4.0863 | 0.0035 | ESPL1 | -2.7033 | 0.0269 |
| PKMYT1 | -4.0707 | 0.0036 | CD1E | -2.7015 | 0.0270 |
| NDC80 | -4.0315 | 0.0038 | CCNB1 | -2.6863 | 0.0277 |
| KIF11 | -3.8926 | 0.0046 | TNKS | -2.6618 | 0.0287 |
| KIF2C | -3.8841 | 0.0046 | ZEB2 | -2.6591 | 0.0288 |
| ASPM | -3.8584 | 0.0048 | PLCB4 | -2.6272 | 0.0303 |
| KIFC1 | -3.787 | 0.0053 | GNLY | -2.6136 | 0.0310 |
| CEP55 | -3.7271 | 0.0058 | NUF2 | -2.6034 | 0.0315 |
| CDC6 | -3.6572 | 0.0064 | MYCT1 | -2.5782 | 0.0327 |
| EXO1 | -3.6365 | 0.0066 | KIF23 | -2.539 | 0.0348 |
| MYBL2 | -3.6101 | 0.0069 | TLR4 | -2.5245 | 0.0356 |
| RAD51 | -3.5547 | 0.0075 | CD8A | -2.5176 | 0.0360 |
| APH1B | 3.5404 | 0.0076 | HLA-B | -2.5011 | 0.0369 |
| DDX39A | -3.4223 | 0.0091 | EIF4E2 | -2.4829 | 0.0380 |
| PLCE1 | -3.2922 | 0.010 | EPAS1 | -2.4789 | 0.0382 |
| NPR1 | -3.2066 | 0.0125 | CREBBP | -2.4786 | 0.0382 |
| ERBB2 | 3.1943 | 0.0127 | TEK | 2.4679 | 0.0388 |
| CTSW | -3.1837 | 0.0129 | CDK1 | -2.4577 | 0.0395 |
| RRM2 | -3.1638 | 0.0133 | SFRP2 | 2.4534 | 0.0397 |
| SMAD1 | -3.0434 | 0.0160 | FOXM1 | -2.4323 | 0.0411 |
| IL12RB2 | -3.0223 | 0.0165 | PIM1 | -2.4179 | 0.0420 |
| PIK3CD | -3.0178 | 0.0166 | PIK3R2 | 2.384 | 0.0443 |
| PARP1 | -3.0123 | 0.0168 | WRN | -2.38 | 0.0445 |
| IL20RA | 2.9819 | 0.0175 | TOP2A | -2.3745 | 0.0449 |
| COLEC12 | -2.9694 | 0.0179 | SPN | -2.3564 | 0.0462 |
| TYK2 | -2.9691 | 0.0179 | E2F1 | -2.3525 | 0.0465 |
| NOTCH2 | -2.9496 | 0.0184 | IL7R | -2.3422 | 0.0473 |
| CXorf36 | -2.9468 | 0.0185 | LTB | -2.3162 | 0.0492 |
| SLC39A6 | 2.9388 | 0.0187 | UBE2C | -2.3126 | 0.0495 |
| BRCA2 | -2.9169 | 0.0194 | IL10RA | -2.3105 | 0.0496 |
| HIST1H2BH | -2.9148 | 0.019 | SOCS1 | -2.3073 | 0.0499 |
| TUBA4A | -2.9022 | 0.0198 | MCM3 | -2.3064 | 0.0500 |
| MFNG | -2.9002 | 0.0199 |  |  |  |

**Supplementary Table 4. Differential genes in peripheral and central tumors in LumB (p<0.05) (pair t-test)**

| Gene | t-stat | *p*-value | Gene | t-stat | *p*-value |
| --- | --- | --- | --- | --- | --- |
| MIS18A | -4.5628 | 0.0004 | FOS | -2.5098 | 0.0250 |
| WIF1 | -4.2179 | 0.0009 | SUV39H2 | -2.5074 | 0.0251 |
| CHEK2 | -4.2045 | 0.0009 | MMRN2 | -2.4918 | 0.0259 |
| EMCN | -3.9343 | 0.0015 | EDN1 | -2.4875 | 0.026 |
| CDK4 | -3.879 | 0.0017 | WDR77 | -2.4769 | 0.0267 |
| RNASE2 | -3.6196 | 0.0028 | PFDN2 | -2.472 | 0.0267 |
| PREP | -3.3881 | 0.0044 | LPL | -2.4699 | 0.0270 |
| SERBP1 | -3.1942 | 0.0065 | SFRP1 | -2.4644 | 0.0273 |
| EGFR | -3.1624 | 0.0069 | FGFR4 | -2.4468 | 0.0282 |
| VEGFR2 | -3.1614 | 0.0069 | CDH5 | -2.4253 | 0.0294 |
| CD36 | -3.0966 | 0.0079 | MET | -2.4103 | 0.0303 |
| PDK4 | -3.0889 | 0.0080 | ZBTB16 | -2.3963 | 0.0311 |
| FGF2 | -2.961 | 0.0103 | CACNA2D3 | -2.3692 | 0.0327 |
| ABCA8 | -2.9564 | 0.0104 | PPP2R1A | -2.347 | 0.0342 |
| JAG2 | -2.9393 | 0.0108 | BBOX1 | -2.3405 | 0.036 |
| ID4 | -2.9388 | 0.0108 | PIK3CA | -2.3261 | 0.0355 |
| ZFYVE9 | -2.9387 | 0.0108 | PLA2G4A | -2.3146 | 0.0363 |
| CLEC14A | -2.8229 | 0.0136 | IL24 | -2.3145 | 0.0363 |
| B3GNT3 | -2.7623 | 0.0153 | CNTFR | -2.3009 | 0.0373 |
| LEMD1 | -2.7531 | 0.0156 | EIF4E2 | -2.2645 | 0.0340 |
| EIF3B | -2.7034 | 0.0171 | NPR1 | -2.2605 | 0.0403 |
| RFC4 | -2.7017 | 0.0172 | MYC | -2.2518 | 0.0409 |
| LEPR | -2.6625 | 0.0186 | MYCT1 | -2.1541 | 0.0491 |
| GSK3B | -2.6384 | 0.0195 | FLT3 | -2.1477 | 0.0497 |
| NUDT1 | -2.6368 | 0.019 | RASGRF2 | 2.1747 | 0.0473 |
| FOXC1 | -2.6161 | 0.0203 | CLDN3 | 2.2406 | 0.0418 |
| GRIN2A | -2.5494 | 0.0231 | SFRP2 | 2.3375 | 0.0348 |
| FGF13 | -2.5414 | 0.0235 | COMP | 2.3964 | 0.0311 |
| TMPRSS2 | -2.5317 | 0.0240 | TFF1 | 2.8544 | 0.0127 |

| 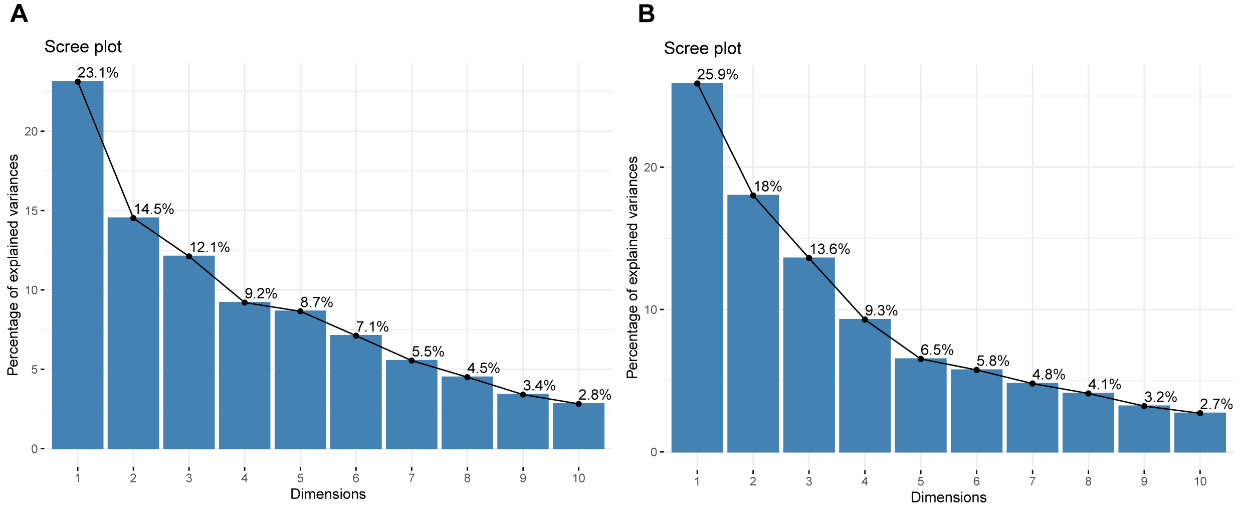 |
| --- |
| **Supplementary Figure 7. Scree plots of eigenvalues after principal component analysis (PCA)** The scree plot shows the percentage of explained variances by the first ten principal components of LumA A) and LumB B) in the center and periphery of the tumor. |

| 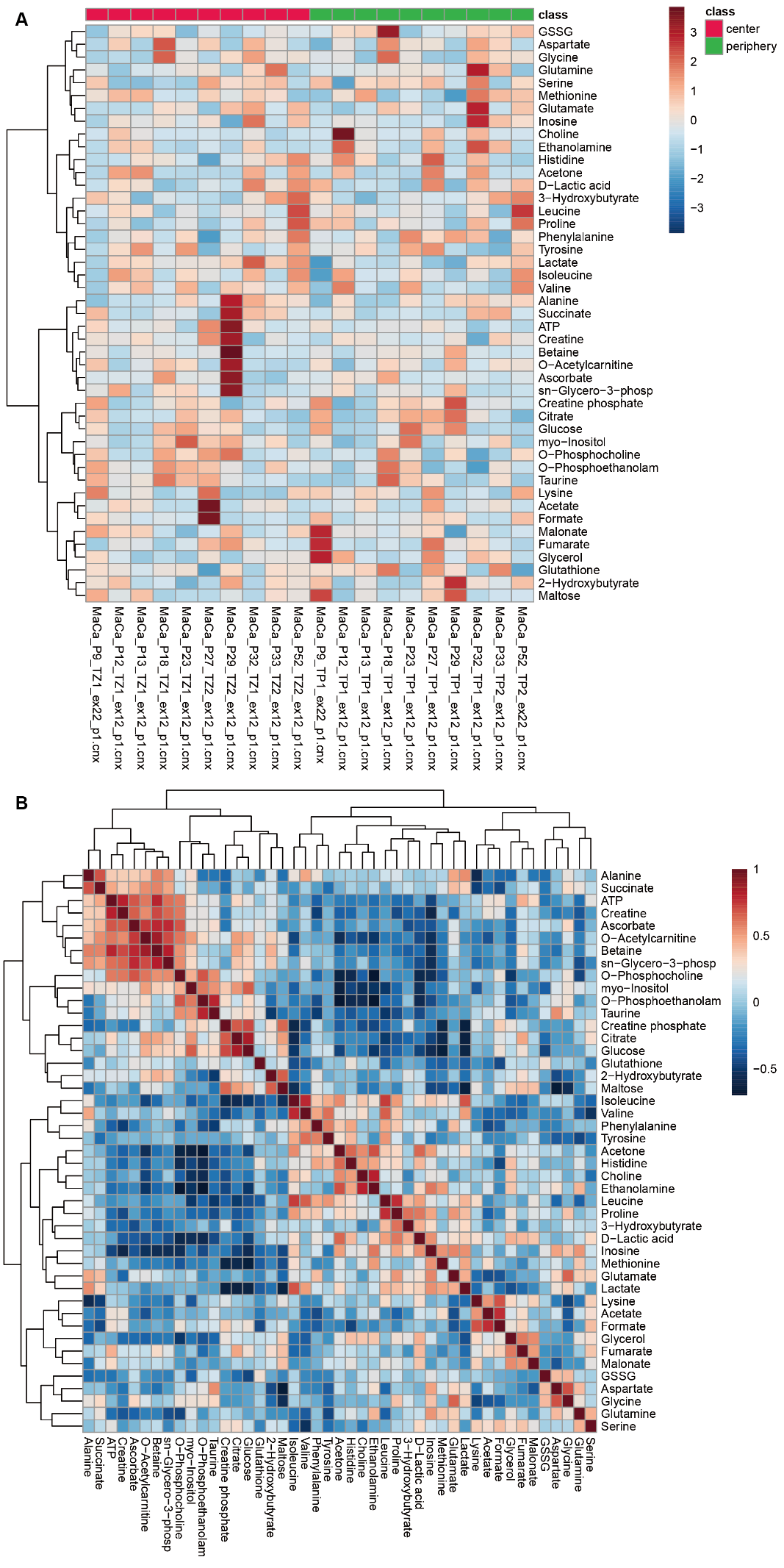 |
| --- |
| 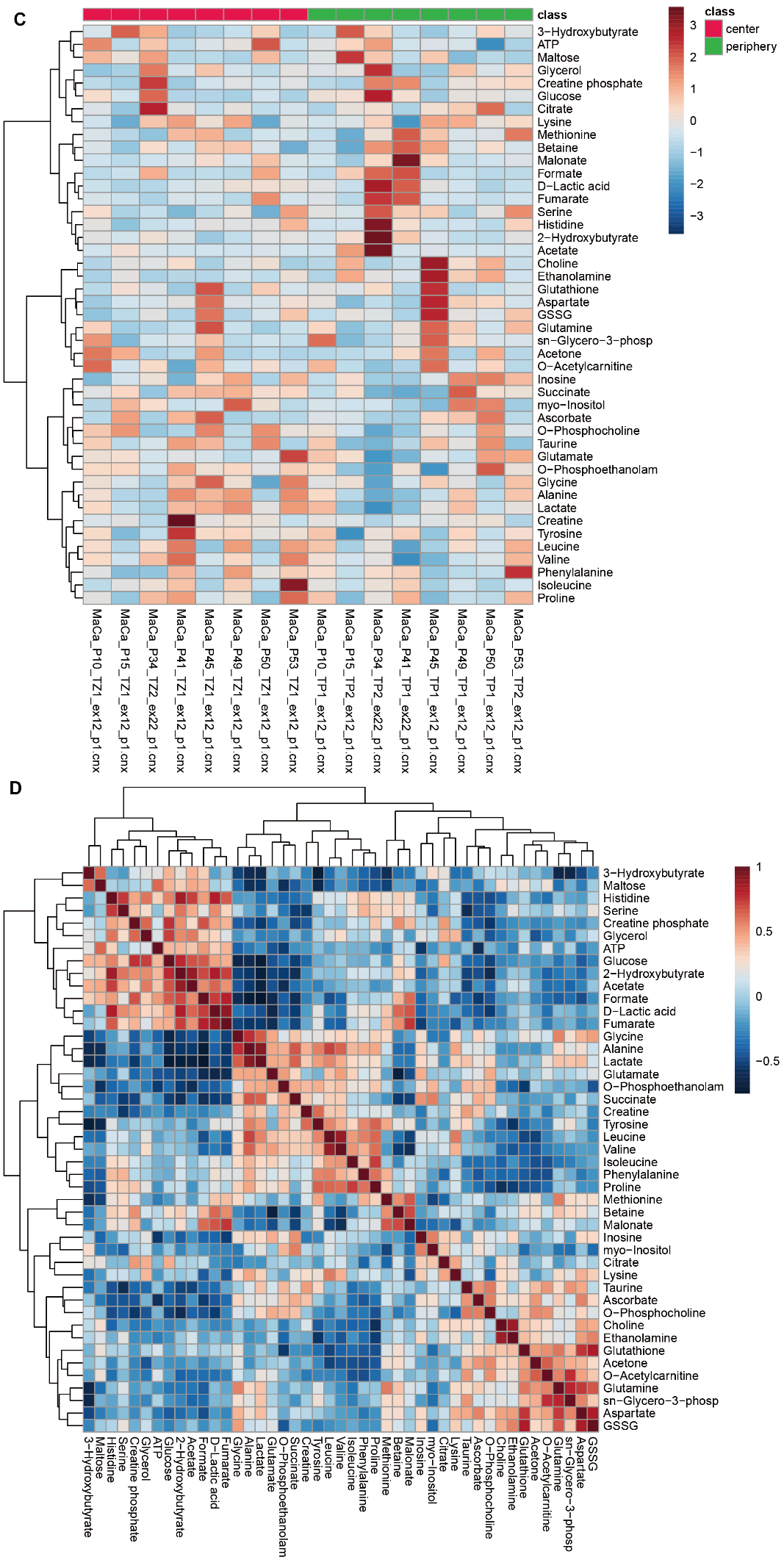 |
| **Supplementary Figure 8. Heatmap and correlation heatmap of LumA and LumB** **tumors** A) Heatmap of all metabolites in LumA tumors. Auto-scaled concentration values are illustrated as red - high, and blue - low concentration. Metabolite list organized by Ward hierarchical clustering using Euclidean distance measure. B) Correlation heatmap of all metabolites in LumA tumors, metabolites were grouped into four clusters according to their Spearman correlation distance. C) Heatmap of all metabolites in LumB tumors. D) Correlation heatmap of all metabolites in LumB tumors, metabolites were grouped into three clusters according to their Spearman correlation distance. |

| 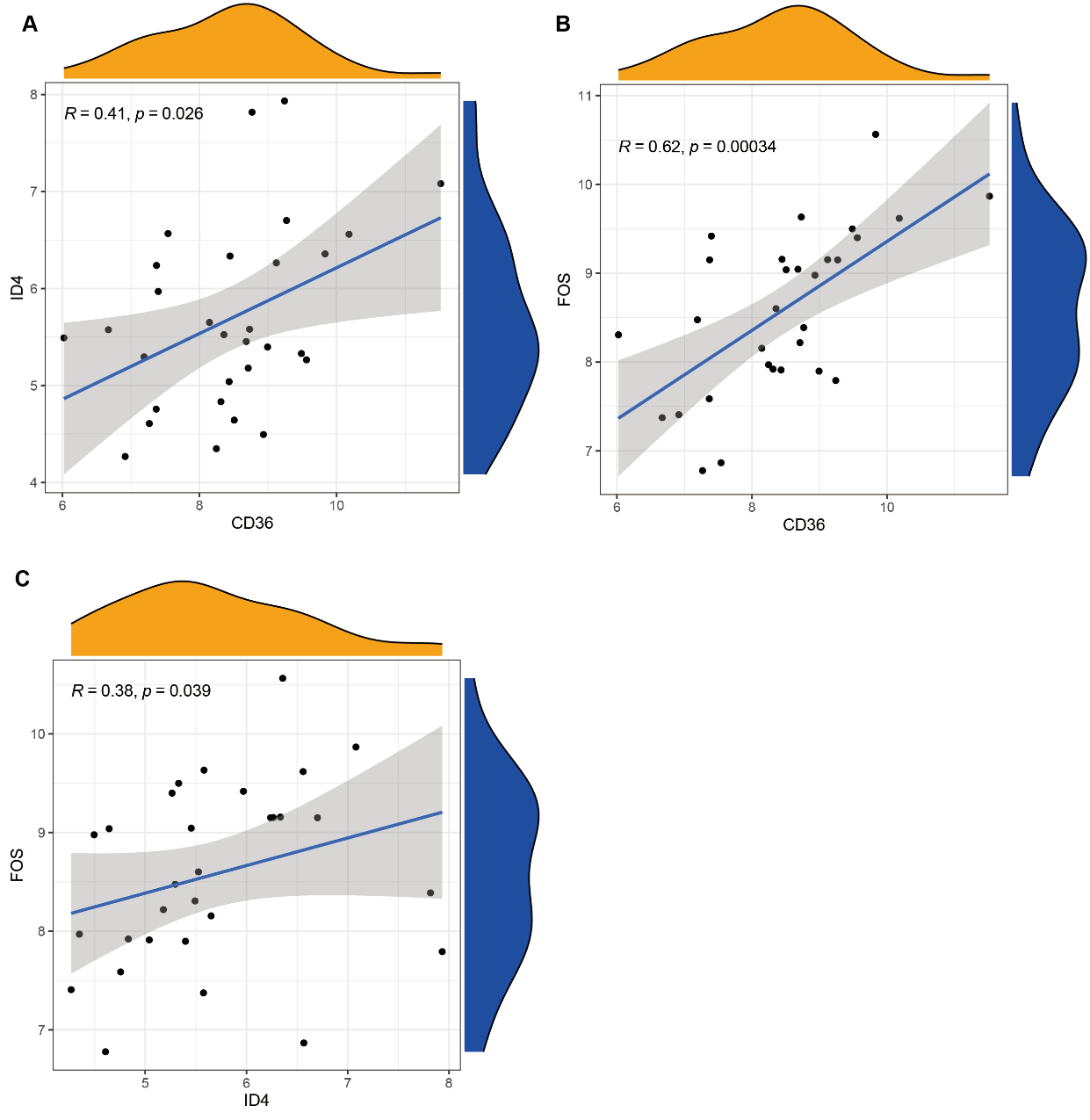 |
| --- |
| **Supplementary Figure 9. Correlations between ID4, FOS, and CD36 in LumB.** Although the correlation between the cluster of differentiation 36 (CD36) and acetate was not significant, CD36 was positively correlated with the Inhibitor of DNA Binding 4 (ID4) and fos proto-oncogene (FOS), which were positively correlated with acetate and indicated the relevance of lipid metabolism in LumB where tumor-surrounding lipid tissue serves as the substrate for tumor growth. |

**Supplementary Table 5. Correlation of central and peripheral significantly different genes with metabolites in LumA.**

| Gene | Metabolites | Correlation | *p*-value | FDR | Regulation |
| --- | --- | --- | --- | --- | --- |
| PLA2G2A | Ascorbate | 0.5967 | 0.0070 | 0.0220 | positive |
| PLA2G2A | Aspartate | 0.5143 | 0.0243 | 0.0275 | positive |
| PLA2G2A | Glutathione disulfide (GSSG) | 0.5203 | 0.0224 | 0.0271 | positive |
| PLA2G2A | Glycine | 0.6967 | 0.0010 | 0.0052 | positive |
| PLA2G2A | O-Phosphocholine | 0.5541 | 0.0138 | 0.0220 | positive |
| PLCE1 | 3-Hydroxybutyrate | 0.5897 | 0.0079 | 0.0220 | positive |
| PLCE1 | Acetate | -0.557 | 0.0133 | 0.0220 | negative |
| PLCE1 | Glutamate | 0.6210 | 0.0045 | 0.0193 | positive |
| PLCE1 | Isoleucine | 0.5068 | 0.0439 | 0.0466 | positive |
| PLCE1 | Leucine | 0.7151 | 0.0006 | 0.0049 | positive |
| PLCE1 | Phenylalanine | 0.5562 | 0.0134 | 0.0220 | positive |
| PLCE1 | Proline | 0.8089 | 2.75E-05 | 0.0004 | positive |
| PLCE1 | Valine | 0.5537 | 0.0139 | 0.022 | positive |
| SLC39A6 | Adenosine triphosphate (ATP) | 0.5084 | 0.0484 | 0.0483 | positive |
| SLC39A6 | Acetate | 0.5229 | 0.0216 | 0.0271 | positive |
| SLC39A6 | Creatine | 0.5407 | 0.0168 | 0.0238 | positive |
| SLC39A6 | Formate | 0.5519 | 0.0143 | 0.0220 | positive |

**Supplementary Table 6. Correlation of central and peripheral significant difference genes with metabolites in LumB.**

| Gene | Metabolites | Correlation | *p*-value | FDR | Regulation |
| --- | --- | --- | --- | --- | --- |
| ABCA8 | Glutamine | 0.5233 | 0.0453 | 0.0479 | positive |
| ABCA8 | Glycine | 0.5777 | 0.0241 | 0.0399 | positive |
| B3GNT3 | Glutamate | -0.611 | 0.0155 | 0.0328 | negative |
| B3GNT3 | Inosine | -0.627 | 0.0123 | 0.0285 | negative |
| B3GNT3 | Succinate | -0.561 | 0.0294 | 0.0414 | negative |
| BBOX1 | Aspartate | 0.5274 | 0.0433 | 0.0476 | positive |
| BBOX1 | Glutathione disulfide (GSSG) | 0.7015 | 0.0036 | 0.0142 | positive |
| BBOX1 | Glutathione | 0.6708 | 0.0062 | 0.0195 | positive |
| BBOX1 | Glycine | 0.5488 | 0.0341 | 0.0423 | positive |
| BBOX1 | Leucine | -0.5450 | 0.0357 | 0.0434 | negative |
| CD36 | Glycine | 0.5524 | 0.0327 | 0.0423 | positive |
| CD36 | Tyrosine | -0.5408 | 0.0374 | 0.0448 | negative |
| CNTFR | Alanine | 0.6721 | 0.0061 | 0.0195 | positive |
| CNTFR | Glycine | 0.7658 | 0.0009 | 0.0062 | positive |
| CNTFR | Lactate | 0.5214 | 0.0462 | 0.0482 | positive |
| CNTFR | Proline | 0.5917 | 0.0201 | 0.0362 | positive |
| COMP | Inosine | -0.6427 | 0.0098 | 0.0242 | negative |
| COMP | Succinate | -0.5538 | 0.0322 | 0.0423 | negative |
| COMP | *myo*-Inositol | -0.6164 | 0.0144 | 0.0323 | negative |
| COMP | sn-Glycero-3-phosphocholine | 0.5771 | 0.0243 | 0.0399 | positive |
| EGFR | Acetone | 0.6321 | 0.0115 | 0.0275 | positive |
| EGFR | Aspartate | 0.5636 | 0.0287 | 0.0412 | positive |
| EGFR | GSSG | 0.7195 | 0.0025 | 0.0112 | positive |
| EGFR | Glutamine | 0.5749 | 0.0250 | 0.0399 | positive |
| EGFR | Glutathione | 0.8634 | 3.38E-05 | 0.0021 | positive |
| EGFR | Isoleucine | -0.5694 | 0.0267 | 0.0423 | negative |
| EGFR | Leucine | -0.6613 | 0.0073 | 0.0208 | negative |
| EGFR | O-Acetylcarnitine | 0.5496 | 0.0338 | 0.0423 | positive |
| EGFR | Phenylalanine | -0.5493 | 0.0340 | 0.0423 | negative |
| EGFR | Proline | -0.5600 | 0.0299 | 0.0414 | negative |
| EGFR | Tyrosine | -0.5268 | 0.0436 | 0.0476 | negative |
| EGFR | Valine | -0.5238 | 0.0450 | 0.0479 | negative |
| FGF2 | Glutathione | 0.5888 | 0.0209 | 0.0367 | positive |
| FGF2 | Phenylalanine | -0.5651 | 0.0281 | 0.0412 | negative |
| FGF2 | Proline | -0.6059 | 0.0166 | 0.0332 | negative |
| FOS | 3-Hydroxybutyrate | 0.7694 | 0.0008 | 0.0062 | positive |
| FOS | Acetate | 0.7234 | 0.0023 | 0.0112 | positive |
| FOS | Alanine | -0.6625 | 0.0071 | 0.0208 | negative |
| FOS | Lactate | -0.7418 | 0.0015 | 0.0092 | negative |
| FOS | Maltose | 0.6465 | 0.0092 | 0.0242 | positive |
| FOS | O-Phosphoethanolamine | -0.5366 | 0.0391 | 0.0461 | negative |
| FOS | Proline | -0.5177 | 0.0481 | 0.0487 | negative |
| FOS | Tyrosine | -0.7976 | 0.0004 | 0.0052 | negative |
| FOS | sn-Glycero-3-phosphocholine | -0.5322 | 0.0411 | 0.0476 | negative |
| ID4 | Acetate | 0.60834 | 0.0161 | 0.0331 | positive |
| ID4 | Glutathione | 0.6806 | 0.0052 | 0.0188 | positive |
| ID4 | O-Phosphoethanolamine | -0.6703 | 0.0063 | 0.0195 | negative |
| MET | GSSG | 0.7377 | 0.0017 | 0.0093 | positive |
| MET | Glutathione | 0.7514 | 0.0012 | 0.0081 | positive |
| MET | Leucine | -0.5755 | 0.0248 | 0.0399 | negative |
| MET | Tyrosine | -0.6024 | 0.0175 | 0.0339 | negative |
| SFRP1 | Acetone | 0.5676 | 0.0273 | 0.0409 | positive |
| SFRP1 | Aspartate | 0.5984 | 0.0184 | 0.0349 | positive |
| SFRP1 | GSSG | 0.7204 | 0.0025 | 0.0112 | positive |
| SFRP1 | Glutamine | 0.6591 | 0.0075 | 0.0208 | positive |
| SFRP1 | Glutathione | 0.7676 | 0.0008 | 0.0062 | positive |
| SFRP1 | O-Acetylcarnitine | 0.5715 | 0.0260 | 0.0407 | positive |
| TFF1 | ATP | 0.5280 | 0.0430 | 0.0476 | positive |
| TFF1 | Glutamate | -0.5267 | 0.0436 | 0.0476 | negative |
| TFF1 | O-Phosphoethanolamine | -0.6427 | 0.0098 | 0.0242 | negative |
| TMPRSS2 | Acetone | 0.5197 | 0.0471 | 0.0484 | positive |
| TMPRSS2 | O-Acetylcarnitine | 0.7121 | 0.0029 | 0.0122 | positive |
| TMPRSS2 | sn-Glycero-3-phosphocholine | 0.6874 | 0.0046 | 0.0175 | positive |
| WIF1 | Acetone | 0.5144 | 0.0498 | 0.0498 | positive |
| WIF1 | Aspartate | 0.8164 | 0.0002 | 0.0049 | positive |
| WIF1 | GSSG | 0.8506 | 5.84E-05 | 0.0021 | positive |
| WIF1 | Glutamine | 0.7983 | 0.0004 | 0.0052 | positive |
| WIF1 | Glutathione | 0.7811 | 0.0006 | 0.0062 | positive |
| WIF1 | Glycine | 0.5959 | 0.0191 | 0.0351 | positive |
| ZFYVE9 | Methionine | 0.5553 | 0.0317 | 0.0423 | positive |
| ZFYVE9 | Succinate | -0.6132 | 0.0151 | 0.0032 | negative |
| ZFYVE9 | *myo*-Inositol | -0.7752 | 0.0007 | 0.0062 | negative |

**Supplementary Table 7. Correlation of one-carbon related genes with serine and other one-carbon metabolites in LumB.**

| Gene | Metabolites | Correlation | *p*-value | FDR | Regulation |
| --- | --- | --- | --- | --- | --- |
| FOXP3 | Serine | -0.5229 | 0.0455 | 0.0904 | negative |
| GDF15 | Serine | -0.5547 | 0.0319 | 0.0830 | negative |
| GRIA3 | Serine | 0.5834 | 0.0224 | 0.0769 | positive |
| LRP2 | Serine | 0.5742 | 0.0252 | 0.0803 | positive |
| MUC1 | Serine | 0.5284 | 0.0429 | 0.0900 | positive |
| FOXP3 | Methionine | -0.5647 | 0.0283 | 0.0815 | negative |
| GDF15 | Glutathione | -0.4524 | 0.0987 | 0.0996 | negative |
| GRIA3 | Glucose | 0.7477 | 0.0014 | 0.0293 | positive |
| GRIA3 | Glutamate | -0.5104 | 0.0519 | 0.0933 | negative |

| 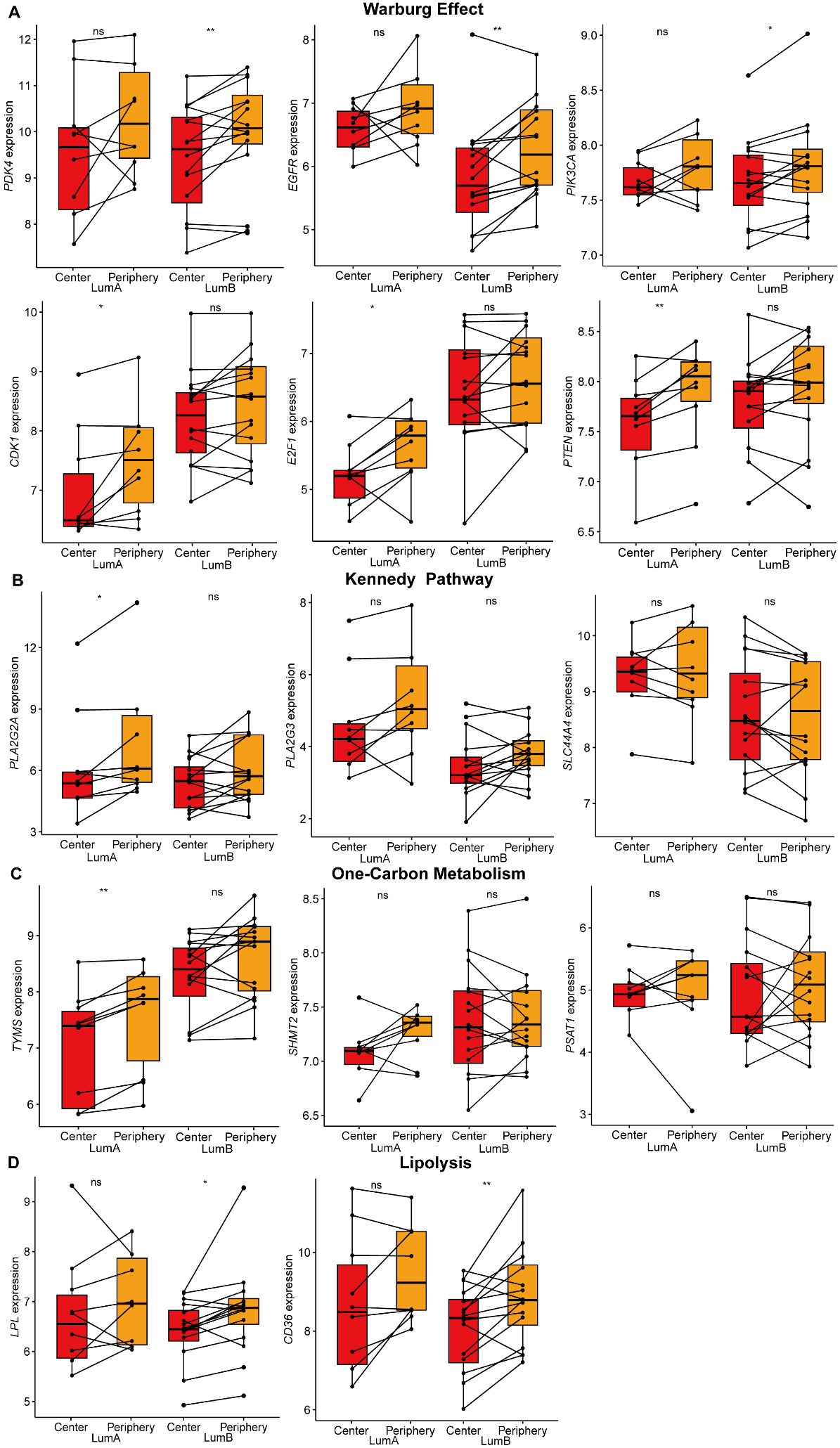 |
| --- |
| **Supplementary Figure 10. Genes associated with metabolic pathways.** A) Warburg effect: PDK4, EGFR, and PIK3CA were significantly increased in the LumB periphery. CDK1, E2F1, and PTEN were significantly increased in the LumA periphery. B) One-carbon metabolism: In the periphery of LumA and LumB, TYMS, SHMT2, and PSAT1 were increased, and TYMS had significant upregulation in the periphery of LumA. C) Kennedy pathway: PLA2G2A, PLA2G3, and SLC44A4 were increased in the periphery of LumA and LumB, and PLA2G2A was significantly upregulated in the periphery of LumA. D) Lipolysis: LPL and CD36 were significantly increased in the LumB periphery. (**p<0.01, *p<0.05) |

| 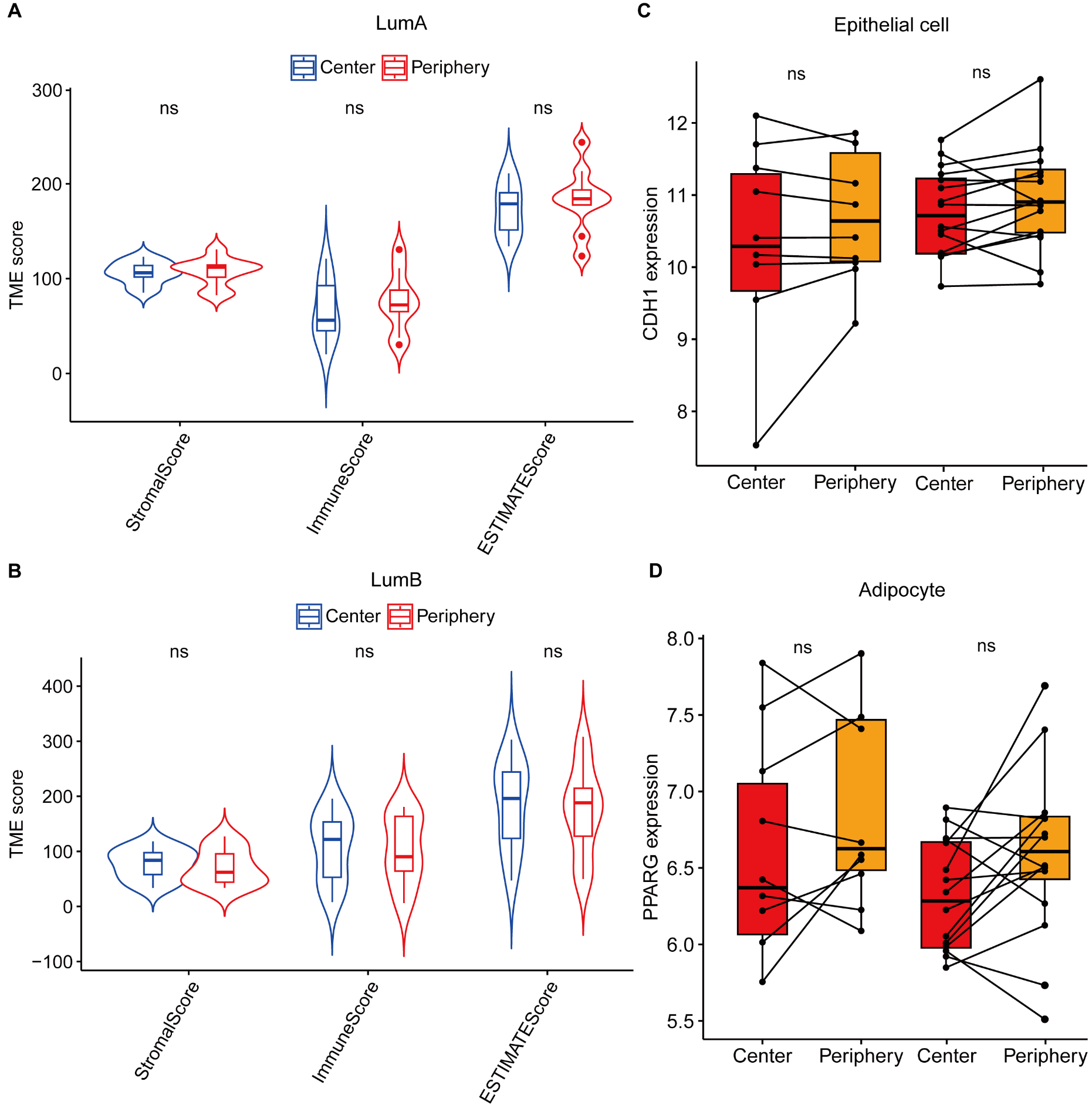 |
| --- |
| **Supplementary Figure 11. Regional cellularity.** The violin diagrams show the stromal score, immune score, and ESTIMATE score in tumor center and periphery of LumA A) and LumB B). The box plots show the cell biomarker genes of epithelial cell C) and adipocyte D). |

| 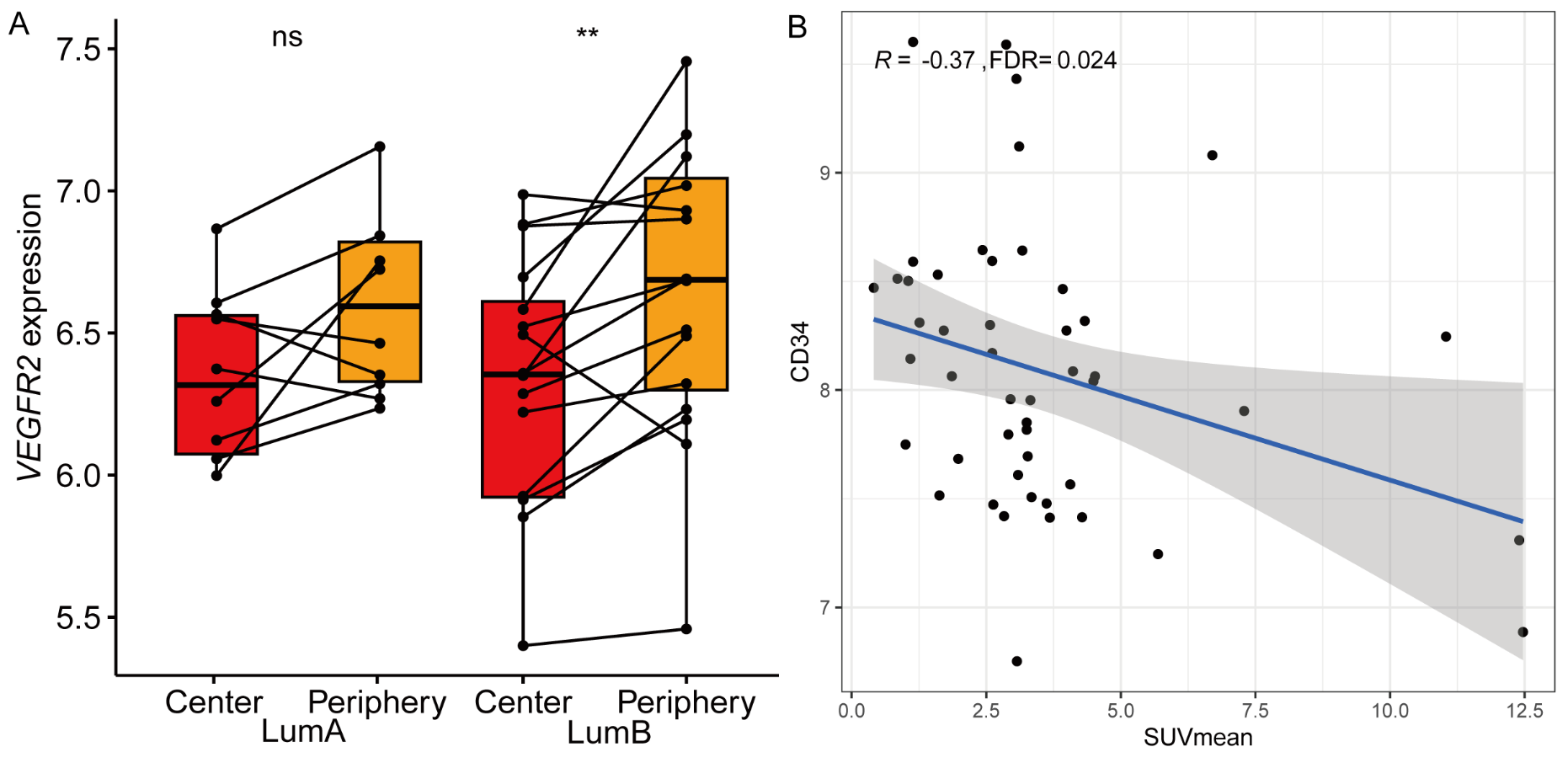 |
| --- |
| **Supplementary Figure 12. Vasculature biomarkers.** Vascular endothelial growth factor receptor 2 (VEGFR2) was increased in the periphery, especially in LumB. (pair t-test, **p<0.01). CD34 Molecule (*CD34*) was negatively correlated with [^18^F]FDG uptake (R=-0.37, FDR<0.05). |
